# Three dimensions of COVID‐19 risk perceptions and their socioeconomic correlates in the United States: A social media analysis

**DOI:** 10.1111/risa.13993

**Published:** 2022-07-13

**Authors:** Shan Qiao, Zhenlong Li, Chen Liang, Xiaoming Li, Caroline Rudisill

**Affiliations:** ^1^ Department of Health Promotion Education and Behavior, Arnold School of Public Health University of South Carolina Columbia South Carolina USA; ^2^ Geoinformation and Big Data Research Lab, Department of Geography University of South Carolina Columbia South Carolina USA; ^3^ Department of Health Services Policy and Management, Arnold School of Public Health University of South Carolina Columbia South Carolina USA; ^4^ South Carolina SmartState Center for Healthcare Quality University of South Carolina Columbia South Carolina USA; ^5^ Big Data Health Science Center University of South Carolina Columbia South Carolina USA

**Keywords:** COVID‐19, risk perceptions, social determinants of health, social media analysis, Twitter data

## Abstract

Social media analysis provides an alternate approach to monitoring and understanding risk perceptions regarding COVID‐19 over time. Our current understandings of risk perceptions regarding COVID‐19 do not disentangle the three dimensions of risk perceptions (perceived susceptibility, perceived severity, and negative emotion) as the pandemic has evolved. Data are also limited regarding the impact of social determinants of health (SDOH) on COVID‐19‐related risk perceptions over time. To address these knowledge gaps, we extracted tweets regarding COVID‐19‐related risk perceptions and developed indicators for the three dimensions of risk perceptions based on over 502 million geotagged tweets posted by over 4.9 million Twitter users from January 2020 to December 2021 in the United States. We examined correlations between risk perception indicator scores and county‐level SDOH. The three dimensions of risk perceptions demonstrate different trajectories. Perceived severity maintained a high level throughout the study period. Perceived susceptibility and negative emotion peaked on March 11, 2020 (COVID‐19 declared global pandemic by WHO) and then declined and remained stable at lower levels until increasing once again with the Omicron period. Relative frequency of tweet posts on risk perceptions did not closely follow epidemic trends of COVID‐19 (cases, deaths). Users from socioeconomically vulnerable counties showed lower attention to perceived severity and susceptibility of COVID‐19 than those from wealthier counties. Examining trends in tweets regarding the multiple dimensions of risk perceptions throughout the COVID‐19 pandemic can help policymakers frame in‐time, tailored, and appropriate responses to prevent viral spread and encourage preventive behavior uptake in the United States.

## 1 INTRODUCTION

The coronavirus disease 2019 (COVID‐19) pandemic caused by SARS‐CoV‐2 has resulted in severe morbidity and mortality and strained healthcare systems across the world. As of May 27, 2022, the cumulative number of coronavirus cases globally exceeded 528 million and over 6.2 million people have died of COVID‐19. The United States has led the world in COVID‐19 fatalities (over 1 million as of May 27, 2022) (Johns Hopkins University and Medicine, 2022). The pandemic has profoundly and adversely altered various aspects of society from health systems and economic growth to individuals’ daily lives, health, and well‐being. Individuals’ behaviors such as complying with preventive measures and vaccination are critical to combat the COVID‐19 pandemic and mitigate its impacts. Risk perceptions would be expected to influence individuals’ preventive and protective behavioral responses to COVID‐19 (Lestari & Ulfiana, 2021; Shmueli, 2021) and evidence thus far suggests that risk perceptions play an important role (Bundorf et al., 2021; de Bruin & Bennett, 2020)

Risk perceptions refer to individuals’ subjective assessments and appraisals regarding the probability of experiencing harms or hazards such as injury, illness, and death. Risk perceptions are often composed of two main dimensions: the cognitive dimension, which is about understanding of risks (e.g., perceived susceptibility and perceived severity), and the emotional dimension, which captures feelings about risks (e.g., fear and dread) (Paek & Hove, 2017). Health behavior theories such as the health belief model (HBM), protection motivation theory (PMT), and the risk perception attitude (RPA) framework emphasize how rational and cognitive aspects of risk perceptions influence health behaviors (Janz & Becker, 1984; Rimal & Real, 2003). Generally, a higher level of perceived susceptibility and perceived severity are related to uptake of protective behaviors and willingness to vaccinate (Agüero, Adell, Giménez, Medina, & Continente, 2011; Prati, Pietrantoni, & Zani, 2011; Rubin, Amlôt, Page, & Wessely, 2009; Rudisill, 2013; van der Weerd, Timmermans, Beaujean, Oudhoff, & van Steenbergen, 2011). With respect to COVID‐19, people who perceive higher risks are more likely to uptake protective behaviors such as handwashing and social distancing (de Bruin & Bennett, 2020). Perceived susceptibility and perceived severity are predictors of COVID‐19 vaccine acceptance and intention to vaccinate (Detoc et al., 2020; Dror et al., 2020; Fisher et al., 2020; Graffigna, Palamenghi, Boccia, & Barello, 2020). In addition, risk perceptions regarding COVID‐19 for others are more predictive of behavioral response than risk perceptions about COVID‐19 for oneself (Sherman et al., 2021).

Emotions can also play an important role in people's experiences and processes of risk assessment. Slovic and colleagues highlighted the tendency to respond based on current emotions when understanding and making judgments about risks. For example, feeling intense dread may make people evaluate a risk as more threatening and prevalent (Slovic, Finucane, Peters, & MacGregor, 2007). Emotional reactions to risks (e.g., fear about the disease) could be independent of cognitive appraisal and act as even stronger determinants of individual perceptions and behaviors (Loewenstein, Weber, Hsee, & Welch, 2001). For example, perceptions of “dread risk” (the risk elicits visceral feelings of terror, uncontrollable, catastrophe, inequality, and uncontrolled) may be more influenced by emotions (Slovic, 1987; Slovic, Fischhoff, & Lichtenstein, 1982; Visschers & Siegrist, 2018; Weber, 2017). In the context of COVID‐19 pandemic, a higher level of fear about COVID‐19 is positively related to vaccine acceptance (Detoc et al., 2020). Similarly, being worried about the health consequences of COVID‐19 was positively associated with the willingness to obey strict hygiene and social distancing restrictions (Sobkow, Zaleskiewicz, Petrova, Garcia‐Retamero, & Traczyk, 2020). Negative emotions (i.e., “sadness,” “fear,” “anger,” and “shock”) associated with COVID‐19 may, however, impede the postitive impact of trust in government on preventive behavior uptake (Min, Shen, Yu, & Chu, 2020).

Social determinants of health (SDOH) are “contextual factors that contribute to increased individual risk of exposure to disease or compromise the ability to protect oneself from infection” (Gupta, Parkhurst, Ogden, Aggleton, & Mahal, 2008, p. 765) (e.g., percentage uninsured, median household income, GINI coefficient, percentage living in poverty, and percentage of high‐school graduates). SDOH disproportionally affect socioeconomically vulnerable communities and populations. Furthermore, populations with higher burdens of SDOH factors, especially some ethnic minority groups in the United States have been at disproportionally greater COVID‐19 risk and experienced worse clinical outcomes. Counties with a larger African American population experienced greater case numbers than counties with a smaller African American population (Zephyrin, Radley, Getachew, Baumgartner, & Schneider, 2020). The national COVID‐19‐related mortality rate for African Americans was 2.4 times higher than that of White Americans (Tracker, 2020). A number of SDOH contribute to health disparities related to COVID‐19 mortality including environmental factors (e.g., air pollution in areas with high African American populations) that could exacerbate lung complications (Brandt, Beck, & Mersha, 2020), occupational risk (e.g., employment types and related exposure) (Hawkins, 2020; Millett et al., 2020), access to care (e.g., lack of insurance and geographic maldistribution of healthcare services) (Moore, Langston, George, & Coughlin, 2020), and structural racism in the healthcare system resulting in biased and suboptimal care (Krouse, 2020; Tan, deSouza, & Raifman, 2021).

A recent literature review on predictors of risk perceptions regarding infectious diseases suggests mixed findings regarding income, employment status, and risk perceptions (Tagini et al., 2021). Generally, people with perceptions of lower financial well‐being may be more likely to report higher perceived risks since they anticipate more financial, cultural, and logistic barriers to adequate medical care and services (Choi, Yoo, Noh, & Park, 2017; Di Giuseppe, Abbate, Albano, Marinelli, & Angelillo, 2008; Jang et al., 2020). However, educational level could be a confounding factor that moderates this relationship because it is related to both socioeconomic status and health literacy/knowledge of a disease (De Zwart et al., 2009; Kim & Kim, 2018). Some studies report that people with higher educational attainment may have a greater perceived risk of a pandemic (Barennes, Harimanana, Lorvongseng, Ongkhammy, & Chu, 2010; Fang, Fang, Tsai, Lan, & Hsu, 2012; Jang et al., 2020). There are also studies showing no significant association between education and risk perceptions (Cui, Liao, Lam, Liu, & Fielding, 2017; Oh, Paek, & Hove, 2015; von Gottberg, Krumm, Porzsolt, & Kilian, 2016; Wang et al., 2018). In the context of the COVID‐19 pandemic, monthly income is positively associated with perceived susceptibility of COVID‐19 (He, Chen, Kong, & Liu, 2021).

Social media data have been characterized as offering real‐time coverage of a large percentage of the population (nearly half of all adults worldwide and two‐third of all American adults use social media) (Perrin & Anderson, 2019) with high volume usage (e.g., approximately 500 million tweets per day on Twitter) (Krikorian, 2013). Social media data (e.g., Twitter data) have been used for infectious disease surveillance and monitoring for some time (Huang, Li, Jiang, Li, & Porter, 2020; Strathdee, Nobles, & Ayers, 2019; Young, Rivers, & Lewis, 2014; Young & Zhang, 2018). It has become a critical source for understanding information exchange and the public's opinions, experiences, and feelings about the COVID‐19 pandemic (Hussain et al., 2021; Kurten & Beullens, 2021). For example, the main topics of COVID‐19‐related English tweets from January to May 2020 included the impact of COVID‐19 on the economy and markets, the spread and growth of COVID‐19 cases, treatment and recovery, the impact on the healthcare sector, and governments’ responses. Sentiment (positive or negative) scores were negative on average for the topics of infection transmission, growth of cases, symptoms, racism, the source of the outbreak, and the political impacts of COVID‐19 (Chandrasekaran, Mehta, Valkunde, & Moustakas, 2020).

Since the start of the COVID‐19 outbreak, researchers have explored the role of social media in disseminating both credible medical information and conspiracies/misinformation (Rosenberg, Syed, & Rezaie, 2020), analyzed public perceptions and attitudes regarding COVID‐19 and COVID‐19 vaccines (Boon‐Itt & Skunkan, 2020; Lyu, Le Han, & Luli, 2021; Yousefinaghani, Dara, Mubareka, Papadopoulos, & Sharif, 2021), and examined trends of risk perceptions based on social media data (Dyer and Kolic, 2020). Dyer and Kolic (2020) developed indicators of risk perception based on emotion and attention presented in tweets from 12 countries between March and June 2020 and compared these indicators with key epidemiological indicators (the number of COVID‐19 confirmed cases and deaths). Twitter users paid great attention to mortality, but with less of an emotional and more of an analytic tone over time. They also found differences across countries in sensitivity to national‐level COVID‐19 mortality figures (Dyer & Kolic, 2020).

Existing work does not, however, adequately address some crucial aspects of using social media data to understand population‐level COVID‐19 pandemic responses. First, existing studies on risk perceptions do not disentangle the three dimensions of risk perceptions (perceived susceptibility, perceived severity, and negative emotion) and investigate them over time. Dimensions of risk perceptions are important to examine separately so that they can be used in design of preventive action and public health messaging. Second, a longer time frame is crucial to understand whether findings come from a discrete moment in time or are mapping the ever‐changing dynamics of the pandemic. For instance, one study in the United States suggested that perceived risks of COVID‐19 infection increased within a 5‐day period in the early stage of the pandemic perhaps as a result of the rapid spread of public health messages (Wise, Zbozinek, Michelini, Hagan, & Mobbs, 2020). Risk perception may vary over time, and may be affected by vaccinations, SARS‐CoV‐2 variants, public health policies, and so forth. There have been no studies illustrating risk perception trajectories and how risk perception may be associated with COVID‐19 epidemiological trends over time. A longer time frame would allow for a better understanding of the nuances in risk perception changes as COVID‐19 evolves. Third, limited studies have included SDOH as potential predictors of risk perceptions. The associations between these structural level factors and risk perceptions of COVID‐19 need further exploration. Particularly, existing work does not integrate SDOH data (e.g., those from county‐level US Census data) with social media data to understand how these factors may affect risk perceptions.

To address these knowledge gaps, we employed a long‐time frame covering 2 years and integrate county‐level SDOH data into analysis. Since the COVID‐19 pandemic continually evolves and there is no well‐accepted definition of its stages at present, for the convenience of describing risk perception trends, we define an “early stage without vaccine” period (January 1, 2020–December 13) and also apply the period definition by the CDC (Iuliano et al., 2022) based on 7‐day moving‐average number of COVID‐19 cases, emergency department visits, hospital admissions, and deaths in the United States (December 14, 2020–January 15, 2022), which roughly posited three peak time periods of the pandemic, that is, Winter 2020–21 Period (December 14, 2020–March 1, 2020), Delta Period (July 15, 2021–November 1, 2021), and Omicron Period (December 15, 2021–January 15, 2022) as well as two stable periods (March 1, 2021–July 15, 2021, and November 1–December 15, 2021, respectively). The current study aims to (1) demonstrate the trajectories of perceived susceptibility, perceived severity, and negative emotion through different periods of COVID‐19 epidemic in the United States based on tweets from January 1, 2020 to December 31, 2021; (2) investigate the degree to which these three trajectories are in accordance with COVID‐19 epidemiological trends (i.e., daily new cases and daily new deaths); and (3) examine the correlations between SDOH and the three dimensions of risk perceptions based on county‐level SDOH data in the United States.

## 2 DATA AND METHODS

### 2.1 Data sources

#### 2.1.1 Geotagged Twitter data

We collected 605,344,419 geotagged tweets within the bounding box enclosing the continental United States posted by over 5,167,534 Twitter users from January 1, 2020 to December 31, 2021 using the public Twitter streaming application programming interface (API) and tweet location filter (Twitter, 2021). The API delivers about 1% random sample of publicly available tweets in real time (Twitter, 2022). As in Martin et al., 2020), we filtered out tweets automatically posted by bots such as weather reports and job offers by checking from which application a tweet was posted (tweet source). Specifically, we manually identified a list of tweet sources from which the tweets are deemed to be posted by a human (not a bot). We then removed all tweets whose sources are not included in the identified source list (Appendix Table 1). A geotagged tweet can be tagged at different spatial resolutions such as exact coordinates, neighborhood, city, or country. We further removed the tweets that are tagged at a spatial resolution lower than a city (e.g., tweets at the state and country level were excluded). After data cleaning and filtering, 502,048,698 geotagged tweets posted by 4,930,130 Twitter users were maintained for further analysis.

#### 2.1.2 COVID‐19 epidemic data and key events

The US national‐level and county‐level daily‐accumulated COVID‐19 confirmed cases and deaths were downloaded from the *New York Times*’ GitHub data repository (New York Times, 2020). The national‐level daily new cases and deaths were derived from the *New York Times*’ data set.

#### 2.1.3 Socioeconomic and demographic data

The US county‐level SDOH and demographic (race/ethnicity) variables were extracted from the 2014–2018 American Community Survey (ACS) 5‐year estimates data (U.S. Census, 2019). The SDOH variables include GINI coefficient (a measure of statistical dispersion aimed to represent the income/wealth inequality within a nation or any other group of people; it ranges from 0 to 1, where zero is perfect equality and one is maximal inequality) (Gini, 1936), median household income, percent unemployed, percent having no health insurance, percent living in poverty, and percent with less than high‐school education. Race/ethnicity variables include percent Black or African American, White, Hispanic or Latino, and Asian. Finally, county‐level population density was also derived from the 2014–2018 ACS data.

### 2.2 Keywords identification for risk perceptions

Three categories of risk perception keywords were identified based on literature, including perceived susceptibility, perceived severity, and negative emotional dimension. Perceived susceptibility captures people's subjective beliefs about how vulnerable and susceptible they are to a disease or other health risk (likelihood or probability of getting the disease). Perceived severity captures how serious people believe a health risk to be and whether it will have adverse physical consequences such as death, disability, and pain, and/or adverse social consequences such as ostracism, stigma, and shame. The emotional dimension depicts how people feel about the risk, such as fear, outrage, dread, and so forth.

We populated laypersons’ words and phrases reflecting these risk perception keywords from standardized vocabularies. This approach has been validated and widely used in identifying health‐related written speech (Zeng & Tse, 2006). Specifically, individual risk perception keywords were identified by two researchers (SQ and CR) in review of the Linguistic Inquiry and Word Count (LIWC), a closed vocabulary of cognitive and emotional terms used by laypersons (Tausczik & Pennebaker, 2010). Keywords that were used during consumer health communications were mapped onto the Ontology of Consumer Health Vocabulary (Amith, Cui, Roberts, Xu, & Tao, 2019), a formal and interoperable semantic web ontology that was developed based on the Consumer Health Vocabulary (CHV) (Zeng & Tse, 2006). Therefore, these identified keywords were confirmed by human experts, standardized by LIWC and CHV, and enhanced in term of generalizability as some could be semantically linked to existing medical/healthcare vocabularies as identified by the Uniformed Medical Language System (UMLS) (Bodenreider, 2004). The final list of key words used to identify the three categories of risk perceptions appears in Table 1.

**TABLE 1:** Identified keywords for the three dimensions of COVID‐19 risk perceptions

Perceived susceptibility (CHV ontology ID)	Perceived severity (CHV ontology ID)	Negative emotion (CHV ontology ID)
Vulnerable/vulnerate Risk/risky Unsafe/not safe (ochv#37555) Suspect Doubt/dubious Hesitate/hesitating Danger/dangerous Unsure Believe/believed Undoubted/undoubting Confused/confusing/confusion Immune /immunity High risk/ high‐risk At risk/ at‐risk Avoid Cancel Postpone	Die Dead/death Lethal Fatal Pain/painful (ochv#9185) Isolate Judge Shame/shameful Suffer/suffering/suffered Paralyzed Restricted	Worse/worthen/worthening Worthened/worst Dread Fear/feared/fearful/fearing (ochv#37463) Scare/scared/scaring (ochv#51823) Outrage Nervous Panic Terrify/terrified/terrifying Worry/worried Anxious/anxiety Stress/stressed Distrust

*Note*: Laypersons’ words and phrases reflecting the risk perception were populated from standardized vocabularies. The identified keywords were confirmed by human experts, standardized by Linguistic Inquiry and Word Count (LIWC) and (Consumer Health Vocabulary) CHV, and enhanced in term of generalizability as some could be semantically linked to existing medical/healthcare vocabularies as identified by the Uniformed Medical Language System (UMLS). A complete ID in CHV ontology is http://sbmi.uth.tmc.edu/ontology/ [identical ID of a concept].

### 2.3 Risk perception indicator (RPI)

We defined the RPI for the three specific risk perception dimensions as the proportion of Twitter users who posted dimension‐specific risk perception tweets among all Twitter users who posted COVID‐19‐related tweets during our study period (Equation 1). The COVID‐19‐related tweets were extracted using the following keywords: coronavirus, covid‐19, covid19, pandemic, epidemic, and virus.

(1) $$\begin{array}{ccc} & & RPI^{*}=\frac{Number\;of\;Twitter\;users\;with\;both\;risk\;perception\;and\;COVID\_19\;keywords}{Number\;of\;Twitter\;users\;with\;COVID\_19\;keywords}.\\ & & {}^{*}\text{Note:}\;\text{there}\;\text{are}\;\text{three}\;\text{RPIs}\;\text{including}\;\text{RPI}\;\text{for}\;\text{perceived}\;\text{susceptibility,}\;\text{perceived}\;\text{severity,}\;\text{and}\;\text{negative}\;\text{emotions.} \end{array}$$

At the national level, a daily RPI was computed for each dimension of risk perceptions from January 1, 2020 to December 31, 2021. The daily RPI was used for temporal analysis to demonstrate the trajectories of each risk perception dimension since the COVID‐19 outbreak in the United States. We calculated an accumulated RPI for each risk perception dimension at the county level by using aggregated Twitter data within the study period. The county‐level accumulated RPIs were used for statistical analysis to examine how SDOH (also county‐level measures) correlate with the three dimensions of risk perceptions.

### 2.4 Data analysis

#### 2.4.1 Temporal trend analysis at the national level

The daily RPI for each of three risk perception dimensions (perceived susceptibility, perceived severity, and negative emotion) were computed and plotted as time series at the national level. This allows us to depict their trajectories since the COVID‐19 outbreak in the United States based on tweets from January 1, 2020 to December 31, 2021. To illustrate the degree to which these three trajectories were in accordance with COVID‐19 pandemic trends, COVID‐19 epidemic data (number of daily new cases and daily new deaths) were overlayed and visually associated with the RPI. Finally, key events since the COVID‐19 outbreak (e.g., initial outbreak, WHO declaration of pandemic, lockdown/stay at home, and reopening) were extracted from news reports and government announcements to examine how these key events affected numbers of posted tweets related to risk perceptions (Appendix Table 2).

#### 2.4.2 Statistical analysis at the county level

To examine how SDOH and demographic variables correlate with attention to the three dimensions of risk perceptions, correlation analysis was performed between the county‐level RPIs and county‐level SDOH variables (GINI coefficient, median household income, percent unemployed, percent with no health insurance, percent living in poverty, and percent with less than high‐school education) and race/ethnicity variables (percent of Black or African American, White, Hispanic or Latino, and Asian). To reduce bias and noise, only counties with more than 100 Twitter users who posted COVID‐19‐related tweets were selected, resulting in 1032 counties being included in the statistical analysis. We further examined the distribution of urbanization status across the 1032 counties using the Urban–Rural Classification Scheme for Counties (i.e., large central metropolitan, large fringe metropolitan, medium metropolitan, small metropolitan, and micropolitan/noncore) (Ingram & Franco, 2014) and compared this distribution with the national level one in 2019 (Appendix Table 3). Spatial distribution of these counties was also demonstrated in a map (Appendix Figure 1).

## 3 RESULTS

### 3.1 Temporal trend of Twitter‐derived risk perceptions

**FIGURE 1:**
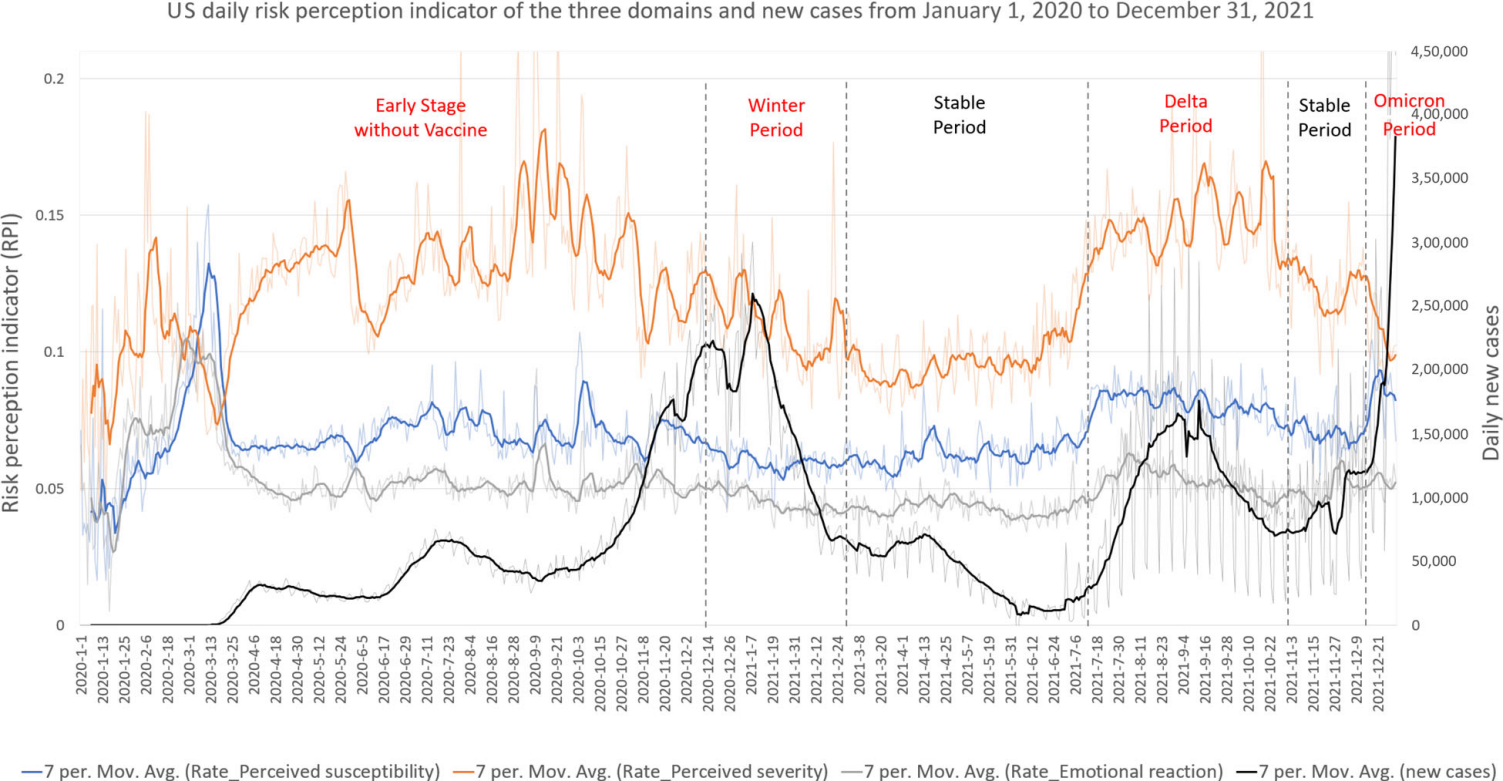
Trajectories for changing trends of three dimensions of COVID‐19 risk perception and COVID‐19 daily new cases *Note*: The scale of RPI (left) is different from the scale of daily new COVID‐19 cases (right). The figure aims to show the trends of different trajectories

Figure 1 illustrates the trajectories for the three dimensions of risk perceptions from January 1, 2020 to December 31, 2021 covering almost three peak periods and two stable periods of the COVID‐19 pandemic in the United States. The trajectories of perceived susceptibility and negative emotion were in accordance with each other displaying great difference to the trajectory of perceived severity. The perceived severity indicator score was generally higher than the ones of perceived susceptibility and negative emotion throughout the whole study period. In addition, the trajectory of perceived severity shows large variation over all periods. The other two trajectories peaked after March 11 when the WHO declared COVID‐19 a global pandemic, then declined quickly (within 1 month) and remained stable for most months. Both trajectories then climbed up slightly during the Delta and Omicron periods with a second peak in perceived susceptibly during the Omicron period but not nearly as high as of March 11, 2020.

**FIGURE 2:**
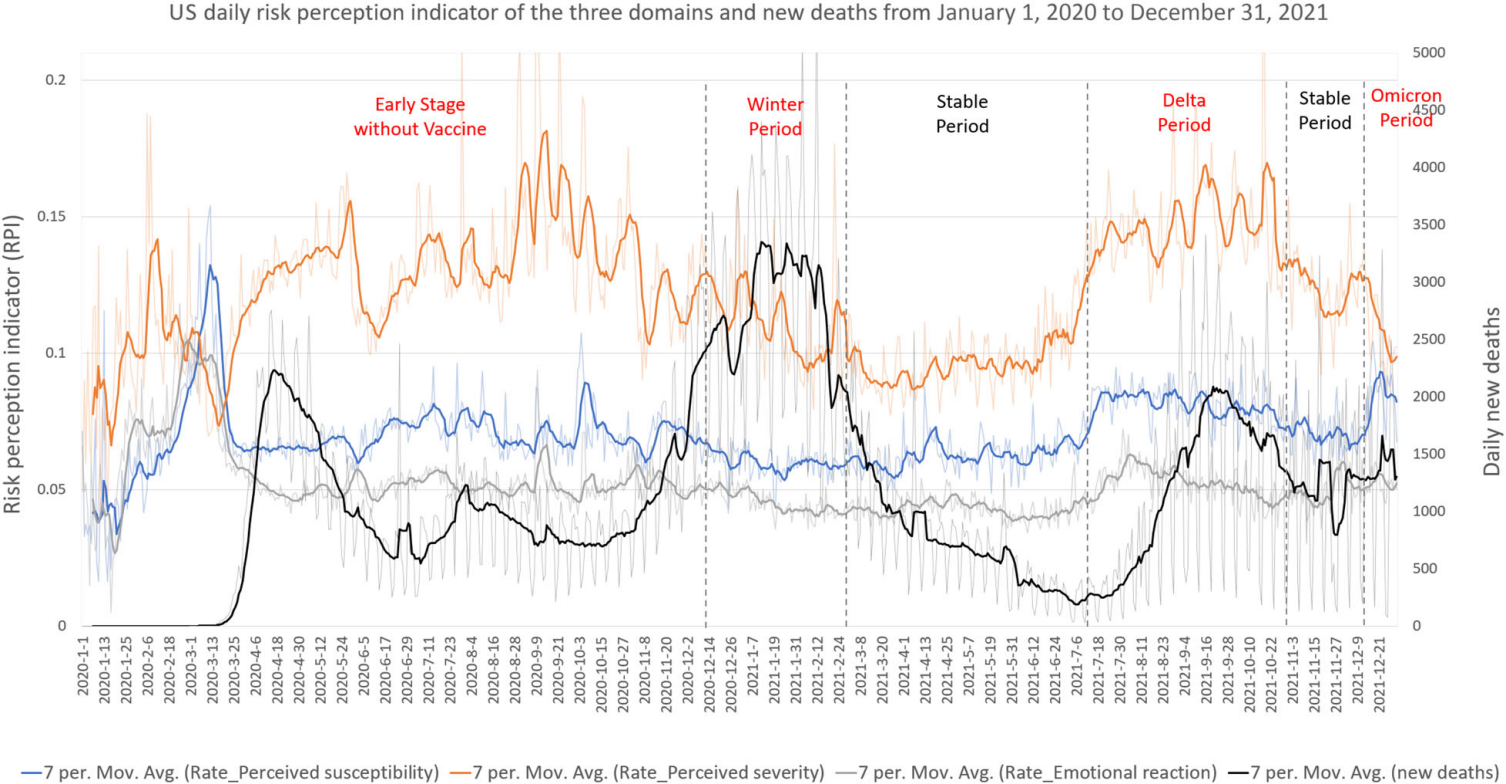
Trajectories for changing trends of three dimensions of COVID‐19 risk perception and COVID‐19 daily new deaths *Note*: The scale of RPI (left) is different from the scale of daily new COVID‐19 deaths (right). The figure aims to show the trends of different trajectories

Comparison between the trajectories of COVID epidemiological indicators (daily new cases and daily new deaths) suggests that the trends in the pandemic's epidemiological status were differentiated from changes in perceptions regarding the COVID epidemic, especially in the first year since the COVID‐19 outbreak (i.e., 2020) in the United States (See Figures 1 and 2). According to CDC data, the peak time of COVID new cases and death cases occurred at the end of 2020, however, the indicator score of perceived severity spiked up on the week of May 28, 2020 when COVID‐19 deaths in the United States passed 100,000. Similarly, the score of perceived susceptibility and negative emotion achieved their maximum levels upon the WHO's declaration of the pandemic when the number of COVID‐19 cases and deaths were very small. In 2021, the trajectories of risk perceptions showed similar trends and generally aligned with COVID‐19 epidemiological trends. It is notable that the temporal trend of Twitter‐derived perceived severity lagged roughly 1 month behind the real‐time change of daily new cases and death cases, while the negative emotions appear 1 month ahead the real epidemiological change.

### 3.2 Correlations between risk perceptions and socioeconomic and demographic factors

Table 2 shows the correlation results between county‐level socioeconomic and demographic variables (e.g., SDOH, race, and population density) and indicator scores of the three risk perception dimensions. In general, low SDOH levels were correlated with low indicator scores of perceived severity and susceptibility. For example, tweets sent from counties with high percentages of the population lacking health insurance, living in poverty, and with education attainment less than high school were significantly related to a low indicator score of perceived susceptibility and severity of COVID‐19 (*p* < 0.01). The high percentage of the population with no health insurance was also significantly linked with a low indicator score of negative emotion (*p* < 0.05). In addition, the indicator score of perceived severity was positively correlated with median household income (*p* < 0.05) and negatively correlated with percentage living in poverty (*p* < 0.01). When examining demographic correlates, we found that a high percentage of Black and Hispanic/Latino people in a county was related to a low indicator score of perceived severity, susceptibility and negative emotion regarding COVID‐19 in Twitter messages, while a high percentage of White people in a county was related to a higher indicator score for all three risk perception dimensions. No significant correlations were observed between the percentage of Asian in a county and the indicator score of any risk perception dimension. There was no significant correlation between the RPI score and county‐level population density.

**TABLE 2:** Correlation results (Pearson correlation coefficient) between county‐level SES and demographic variables and the indicator score for three dimensions of COVID risk perception (number of counties *N* = 1032)

Variable	Perceived susceptibility	Perceived severity	Negative emotion
GINI coefficient	−0.0069	−0.0906**	−0.0404
Median household income	0.0551	0.0941**	−0.0169
Percentage of being unemployed	−0.0362	−0.0233	0.0110
Percentage of no health insurance	−0.2040**	−0.1574**	−0.0707*
Percentage of living in poverty	−0.0860**	−0.1798**	−0.0335
Percentage of less high school	−0.1844**	−0.1727**	−0.0411
Percentage of African American	−0.1765**	−0.2002**	−0.2051**
Percentage of White	0.1570**	0.1428**	0.1809**
Percentage of Hispanic/Latino	−0.1240**	−0.0643*	0.0001
Percentage of Asian	0.0238	0.0236	−0.0117
Population density	0.0008	0.0130	−0.0129

*Notes*: Only counties with more than 100 Twitter users who posted COVID‐19‐related tweets were selected, yielding 1032 counties being included in the statistical analysis. The distribution of urbanization status of these counties was illustrated (Appendix Figure 1) and compared with the national level distribution in 2019 (Appendix Table 3).

* *p *< 0.05;

** *p *< 0.01.

## 4 DISCUSSION

Using over 502 million geotagged tweets posted by over 4.9 million Twitter users from January 1, 2020 to December 31, 2021 in the United States, we extracted tweets regarding risk perceptions related to COVID‐19 and developed indicators on three dimensions (i.e., perceived severity, susceptibility, and negative emotion). We demonstrated and compared the trajectories of the three dimensions with the COVID‐19 epidemic trend during a 2‐year time frame covering different periods of the pandemic. In addition, we investigated how county‐level SDOH and demographic factors correlated with the three dimensions of COVID‐19 risk perceptions.

### 4.1 Temporal patterns of different risk perception dimensions

The three dimensions of risk perceptions demonstrate different trajectories. Generally, Twitter users were more concerned about the severity of COVID‐19 than their perceived susceptibility or associated negative emotions as the indicator score of perceived severity was much higher than the ones of others throughout the study duration. Conversely, the indicator scores for perceived susceptibility and negative emotion declined and remained stable at a lower level after peaks on March 11 when COVID‐19 was declared a global pandemic by the WHO, then they slightly increased during the Delta and Omicron Periods. Perceived susceptibility and negative emotion did, however, have very similar patterns both in level and trajectory.

The high‐level score of perceived severity implies Twitter users’ growing awareness of COVID‐19 as related scientific findings emerged. However, an increased attention to severity over time might not contribute to more discussion about perceived susceptibility. These results seem to be aligned with existing studies. For example, a survey of 1591 people in the United States each day over the first week of the pandemic found growing awareness of general COVID‐19 risk but underestimated personal susceptibility of infection relative to the average person in the United States (Wise et al., 2020). A six‐wave repeated cross‐sectional survey of 1942 participants in China between February 7 and April 23, 2020 also showed an increasing perceived severity but slightly declining perceived susceptibility over time (Rui, Yang, & Chen, 2021). Optimism bias, which is associated with the belief that we are less likely to get an infection or a disease than others, may be one reason and has appeared across a variety of health‐related contexts (Branstrom & Brandberg, 2010; Kuper‐Smith, Doppelhofer, Oganian, Rosenblau, & Korn, 2020). One longitudinal study (a two‐wave telephone survey) among 588 predominately older adults with at least one chronic condition recruited in Chicago suggested that although participants increasingly perceived COVID‐19 as a serious health threat from Wave 1 survey (March 13–20, 2020) to Wave 2 survey (March 27–April 7, 2020), the proportion of participants who believed they were not at all likely to get infected only slightly decreased (Bailey et al., 2020).

The indicator score of negative emotion remained stable at a lower level across the periods after a peak when the WHO declared COVID‐19 a global pandemic. There are several potential explanations. First, many people started feeling fatigued (pandemic fatigue) due to continual exposure to COVID‐19‐related reporting (WHO Regional Office for Europe, 2020). As the pandemic evolved, they might become numb with the news and reduce the frequency in posting or reposting on Twitter about negative feelings (Rypdal, Bianchi, & Rypdal, 2020). Second, the low level of the indicator score for negative emotions was consistent with that of perceived susceptibility. One study conducted in Israel suggests that low perceived susceptibility of COVID‐19 risk significantly buffered the impact of perceived poor health status on emotional reactions toward COVID‐19 (Inbar & Shinan‐Altman, 2021). Third, COVID‐19, certainly in the beginning of 2020 and over 2020 had been an “invisible risk” rather than “dread risk” for the public (Savadori & Lauriola, 2021). According to existing psychological theories on risk perception, “invisible risk” is not as closely associated with emotions as “dread risk” is (Savadori & Lauriola, 2021). To some extent, the number of COVID‐19 cases does not always evoke strong emotions or feelings. In addition, many people have difficulties in understanding numerical information related to risk (Cokely, Galesic, Schulz, Ghazal, & Garcia‐Retamero, 2012). Finally, positive coping strategies, resilience, and social support might help people to bounce back from various negative emotions during the pandemic (Masten & Motti‐Stefandi, 2020). For example, sentiment analysis based on COVID‐19‐related tweets from January to May 2020 suggested a reversal of sentiments from negative to positive for topics such as public prevention, government response, impact on healthcare industry, and COVID‐19 treatment and recovery (Chandrasekaran et al., 2020).

### 4.2 Risk perceptions and epidemiological trends

In addition to demonstrating temporal patterns of different risk perception domains, we further compared risk perception trajectories and COVID‐19 epidemiological trends over time.

The relationship between risk perception trajectories and epidemiological trends shows different dynamics in the 2 years. In year 1 of the pandemic (2020), in contrast to assumptions that actual risk (i.e., COVID‐19 prevalence rate) may play a factor in perceived risk, attentions/discussions on perceptions of COVID‐19 risk did not always follow trends in COVID‐19 epidemiological indicators (e.g., daily new cases and daily new deaths). However, they did appear to be triggered by big news or events (e.g., on May 28, 2020, COVID‐19 deaths in the United States passed 100,000). This finding aligns with studies from the early stages of the COVID‐19 pandemic, which reported that public response was very sensitive to significant social events (Han, Wang, Zhang, & Wang, 2020). News coverage and media exposure have shaped people's risk perceptions (Tsoy, Tirasawasdichai, & Kurpayanidi, 2021; Zeballos Rivas et al., 2021).

In year 2 (2021), risk perception trajectories were generally in accordance with the epidemiological trends based on new cases and death cases, especially in the Delta and Omicron periods. This dynamic may imply that people have been adjusting to the pandemic and learning to assess the risk/severity in a more rational way (i.e., based on the epidemic data) after the fear and panic due to uncertainty of an infectious disease. Twitter users’ attention and discussion of risks may be delayed upon receipt of new information. According to the Diffusion of Innovation Theory, the dissemination of new information and knowledge among populations may need time. Similarly, development of risk perceptions about COVID‐19 could also be a procedure of access, uptake, and interpretation of information about this new virus.

It is notable, however, that trajectory of perceived severity seemed to lag the trend of COVID‐19 cases and deaths, while trajectory of negative emotions was ahead of COVID‐19 cases and death trends. This differentiation in timing further highlights heterogeneity across risk perception domains in response to COVID‐19. Emotionally driven messages may spread faster than those driven by scientific data or empirical evidence, especially in the presence of social media (Brady et al., 2017). Negative emotions particularly facilitate the diffusion of information (Zhu, Kim, & Park, 2020), which may partly explain why negative emotions peaked prior to increases in epidemiological markers. While more evidence is needed to explore the potential “lagging effect” between COVID‐19 epidemiological updates (e.g., daily new cases and death cases) and perceived severity, policymakers and health educators need to realize this potential lag. This lag is important to avoid underestimating difficulties in disseminating accurate information and promoting protective behaviors within a short term.

Our findings demonstrate that perception and understanding of risks regarding a new public health threat is complicated and evolving. In addition, people's attention to multiple dimensions of risk perceptions may show different patterns. Policymakers and public health professionals need to consider and monitor multiple dimensions of risk perception through different pandemic time periods. Health communication and education interventions can be tailored along with the evolution of the public health emergency. Social marketing and health communication campaigns are needed to communicate using effective alerts and reminder messages when people have “prevention fatigue.”

### 4.3 SDOH and risk perceptions

Risk perceptions were not just shaped by COVID‐19 epidemiological trends but also other contextual factors such as the SDOH of communities. Our study suggests that SDOH such as income, race, education level, poverty, and health insurance were correlated with discussions regarding risk perceptions. Twitter users from socioeconomically vulnerable counties showed lower attention on perceived severity and susceptibility of COVID‐19. Low educational attainment could lead to low health literacy, which increases difficulties in understanding health information (Friis, Lasgaard, Rowlands, Osborne, & Maindal, 2016; Paakkari & Okan, 2020). People with lower health literacy were more likely to report less perceived susceptibility to COVID‐19 (Bailey et al., 2020). African Americans, influenced by their cultural contexts and health beliefs and a history of distrust in the health system (Boulware, Cooper, Ratner, LaVeist, & Powe, 2016), might underestimate their infection risk (Bailey et al., 2020; Eiser & Ellis, 2007; Paakkari & Okan, 2020). For example, a study on COVID‐19‐related tweets posted by African Americans (*n* = 1763) from January 21 to May 3, 2020 reported that positive sentiments and optimism were uniquely observed in African American Twitter communities. The percentage of topics like “Black strong” (27.1%) and “growing up Blacks” (22.8%) was higher than COVID‐19 prevention behaviors such as encouraging social distancing (9.4%) and masks wearing (4.7%) (Odlum et al., 2020). Another potential attributor of the low attention to COVID‐19 risk is the existing everyday challenges faced by this population. They paid more attention to urgent needs regarding food security, finances, and racism compared to COVID susceptibility (Odlum et al., 2020).

Our results show a significant correlation between SDOH and the level of attention/discussion regarding perceived severity and susceptibility of COVID‐19 among Twitter users. This disparity of COVID‐19 risk perceptions aligns with the great health disparities seen in COVID‐19 case rates and clinical outcomes in the United States (Loomba et al., 2021; Okonkwo et al., 2020; Zhang & Schwartz, 2020). Given that people from counties with low SDOH may be more likely to be exposed to the virus because of their working conditions (e.g., they cannot work at home), low awareness of risk could increase their vulnerability toward COVID‐19. These groups should be prioritized for health education and promotion receiving tailored messages using understandable and culturally appropriate language.

### 4.4 Limitations, implication, and future studies

This study has several methodological limitations that require attention in interpreting and generalizing the findings. First, we need to be cautious about the representativeness of Twitter users. Twitter is not universally used in the United States, particularly among older and low‐income populations. In addition, not all Twitter users share their geolocation information. Therefore, those who geotag their tweets may not be representative of the wider Twitter population (Jiang, Li, & Ye, 2019). Similarly, some rural counties may not be included in our data analysis due to the small number their geotagged tweets (as shown in Appendix Table 3). Therefore, the current study based on Twitter data does not take the place of survey‐based studies using a representative sample of the population but provides a supplementary approach to explore similar research topics and examine robustness of findings across methods. Second, we used indicator scores as proximal indicators to quantify people's attention (relative frequency of tweet posts) to the three dimensions of the risk perceptions. We did not use existing validated instruments to assess the level of perceived severity, susceptibility, or negative emotions. Third, the keyword‐based tweets retrieval method may miss a small number of relevant tweets that did not include common language regarding risk perceptions. Specifically, keywords‐based methods only capture tweets with an exact match of terms. Indirect mentions of risk‐perception terms and subtle cues may be missed because human natural language is rich and dynamic. Text‐mining methods, such as topic modeling would supplement to further strengthen the understanding of people's opinions. Fourth, in terms of emotion, we only examined negative emotional reaction to COVID‐19 in the analysis. Self‐efficacy and resilience could be other dimensions of risk perceptions (Jahangiry et al., 2020). Further studies are needed to investigate the trend of positive emotional reactions over time during the pandemic. Finally, limited by the scope of the current study, we were not able to elaborate the trajectories of RPI score during the COVID‐19 pandemic within county level because we used an accumulated indicator score for each county. This limits our examination of how perceived risk perceptions change within a county over time and how this change may be affected by SDOH. Although we conducted correlation analysis between aggregated RPIs and county‐level SDOH variables, we still need to be cautious in interpreting results since social media data and national‐level survey data differ across many features.

Despite these limitations, the current study suggests that social media analysis integrated with geospatial data can be a promising tool for real‐time monitoring of risk perceptions during a new public health threat. Examination of changing trends in tweets regarding multiple dimensions of risk perceptions throughout the COVID‐19 pandemic can help governments, policymakers, and healthcare agencies frame in‐time, tailored, and appropriate responses to prevent the pandemic's spread in the United States. Living in a county with relatively low SDOH was correlated with a low level of attention to perceived severity and susceptibility of COVID‐19. Communities with low SDOH and high percentages of African Americans need to be prioritized in health communication campaigns and interventions. Key messages in social marketing and health promotion should be tailored in accordance with patterns of changing trends about risk perceptions throughout the pandemic.

To further advance our understanding regarding the complexity of risk perceptions and its implications in the COVID‐19 context, this work could be expanded in several ways. First, future work can explore the association of risk perception and COVID mitigation measures and behaviors. For example, examining the interaction between risk perceptions from tweets, COVID prevention behaviors, and other potential confounding factors such as politics attitudes (e.g., support Trumps in 2020 election) would provide more insight into factors that impact risk perceptions and behaviors. Second, machine‐learning algorithms for content analysis of extracted COVID‐19‐related tweets can assist in understanding the main themes around COVID‐19 risks. In addition, there is work to be undertaken exploring how people interact with others when presenting risk perceptions in posts.

## 5 CONCLUSION

Risk perception about a new public health threat such COVID‐19 is complex and evolves in ways that may not be accordant with epidemiological trends. People's attention may also be differentiated across multiple dimensions of risk perceptions (e.g., severity, susceptibility, and negative emotions). SDOH such as income, race, education level, poverty, and health insurance were correlated with public discourse on Twitter regarding risk perceptions. Socioeconomically disadvantaged communities, unfortunately, may be overly optimistic or giving less attention to perceived severity and susceptibility of COVID‐19 because of competing everyday demands. These findings are crucial to inform effective intervention strategies for COVID‐19 vaccine administration, prevent COVID‐19 further outbreaks and handle other public health crises in future. More empirical studies using other data sources and novel analysis approaches are needed to advance our understanding of risk perception theory as it relates to new risks such as COVID‐19.

## CONFLICT OF INTEREST

The authors declare that there is no conflict of interest that could be perceived as prejudicing the impartiality of the research reported.

## Supporting information


**Appendix Table 1**. Twitter sources that indicate non‐human‐posted tweets selected by manual checking
**Appendix Table 2**. Key events regarding COVID‐19 pandemic from January 2020 to December 2021
**Appendix Table 3**. Urban‐rural distribution of the selected counties (with Twitter users greater than 100) based on the 2013 National Center for Health Statistics (NCHS) Urban‐Rural Classification Scheme for Counties

## References

[risa13993-bib-0001] Agüero, F. , Adell, M. N. , Giménez, A. P. , Medina, M. J. L. , & Continente, X. G. (2011). Adoption of preventive measures during and after the 2009 influenza A (H1N1) virus pandemic peak in Spain. Preventive Medicine, 53(3), 203–206. 21781983 10.1016/j.ypmed.2011.06.018 PMC7119352

[risa13993-bib-0002] Amith, M. , Cui, L. , Roberts, K. , Xu, H. , & Tao, C. (2019). Ontology of consumer health vocabulary: Providing a formal and interoperable semantic resource for linking lay language and medical terminology [Conference presentation]. IEEE International Conference on Bioinformatics and Biomedicine (BIBM). 10.1109/bibm47256.2019.8983220 PMC10732699 38125584

[risa13993-bib-0003] Bailey, S. C. , Serper, M. , Opsasnick, L. , Persell, S. D. , O'Conor, R. , Curtis, L. M. , Benavente, J. Y. , Wismer, G. , Batio, S. , Eifler, M. , Zheng, P. , Russell, A. , Arvanitis, M. , Ladner, D. P. , Kwasny, M. J. , Rowe, T. , Linder, J. A. , & Wolf, M. S. (2020). Changes in COVID‐19 knowledge, beliefs, behaviors, and preparedness among high‐risk adults from the onset to the acceleration phase of the US outbreak. Journal of General Internal Medicine, 35, 3285–3292. 32875509 10.1007/s11606-020-05980-2 PMC7462357

[risa13993-bib-0004] Barennes, H. , Harimanana, A. N. , Lorvongseng, S. , Ongkhammy, S. , & Chu, C. (2010). Paradoxical risk perception and behaviours related to Avian Flu outbreak and education campaign, Laos. BMC Infectious Diseases, 10(1), 1–7. 20937155 10.1186/1471-2334-10-294 PMC2959065

[risa13993-bib-0005] Brady, W. J. , Wills, J. A. , Jost, J. T. , Tucker, J. A. , & Van Bavel, J. J. (2017). Emotion shapes the diffusion of moralized content in social networks. Proceedings of the National Academy of Sciences, 114(28), 7313–7318. 10.1073/pnas.1618923114 PMC5514704 28652356

[risa13993-bib-0006] Bodenreider, O. (2004). The unified medical language system (UMLS): Integrating biomedical terminology. Nucleic Acids Research, 32(suppl_1), D267–D270. 14681409 10.1093/nar/gkh061 PMC308795

[risa13993-bib-0007] Boon‐Itt, S. , & Skunkan, Y. (2020). Public perception of the COVID‐19 pandemic on Twitter: Sentiment analysis and topic modeling study. JMIR Public Health and Surveillance, 6(4), e21978. 33108310 10.2196/21978 PMC7661106

[risa13993-bib-0008] Boulware, L. E. , Cooper, L. A. , Ratner, L. E. , LaVeist, T. A. , & Powe, N. R. (2016). Race and trust in the health care system. Public Health Reports, 118(4), 358–365. 10.1016/S0033-3549(04)50262-5 PMC1497554 12815085

[risa13993-bib-0009] Brandt, E. B. , Beck, A. F. , & Mersha, T. B. (2020). Air pollution, racial disparities, and COVID‐19 mortality. Journal of Allergy and Clinical Immunology, 146(1), 61–63. 10.1016/j.jaci.2020.04.035 32389591 PMC7204717

[risa13993-bib-0010] Branstrom, R. , & Brandberg, Y. (2010). Health risk perception, optimistic bias, and personal satisfaction. American Journal of Health Behavior, 34(2), 197–205. 19814599

[risa13993-bib-0011] Bundorf, M. K. , DeMatteis, J. , Miller, G. , Polyakova, M. , Streeter, J. L. , & Wivagg, J. (2021). Risk perceptions and protective behaviors: Evidence from COVID‐19 pandemic (No. w28741). National Bureau of Economic Research.

[risa13993-bib-0012] Chandrasekaran, R. , Mehta, V. , Valkunde, T. , & Moustakas, E. (2020). Topics, trends, and sentiments of tweets about the COVID‐19 pandemic: Temporal infoveillance study. Journal of Medical Internet Research, 22(10), e22624. 10.2196/22624 33006937 PMC7588259

[risa13993-bib-0013] Choi, D.‐H. , Yoo, W. , Noh, G.‐Y. , & Park, K. (2017). The impact of social media on risk perceptions during the MERS outbreak in South Korea. Computers in Human Behavior, 72, 422–431. 32288176 10.1016/j.chb.2017.03.004 PMC7126097

[risa13993-bib-0014] Cokely, E. T. , Galesic, M. , Schulz, E. , Ghazal, S. , & Garcia‐Retamero, R. (2012). Measuring risk literacy: The Berlin numeracy test. Judgment and Decision Making, 7(1), 25–47.

[risa13993-bib-0015] Cui, B. , Liao, Q. , Lam, W. W. T. , Liu, Z. P. , & Fielding, R. (2017). Avian influenza A/H7N9 risk perception, information trust and adoption of protective behaviours among poultry farmers in Jiangsu Province, China. BMC Public Health, 17(1), 1–13. 28521760 10.1186/s12889-017-4364-y PMC5437685

[risa13993-bib-0016] De Bruin, W. B. , & Bennett, D. (2020). Relationships between initial COVID‐19 risk perceptions and protective health behaviors: A national survey. American Journal of Preventive Medicine, 59(2), 157–167. 32576418 10.1016/j.amepre.2020.05.001 PMC7242956

[risa13993-bib-0017] De Zwart, O. , Veldhuijzen, I. K. , Elam, G. , Aro, A. R. , Abraham, T. , Bishop, G. D. , Voeten, H. A. , Richardus, J. H. , & Brug, J. (2009). Perceived threat, risk perception, and efficacy beliefs related to SARS and other (emerging) infectious diseases: Results of an international survey. International Journal of Behavioral Medicine, 16(1), 30–40. 19125335 10.1007/s12529-008-9008-2 PMC2691522

[risa13993-bib-0018] Detoc, M. , Bruel, S. , Frappe, P. , Tardy, B. , Botelho‐Nevers, E. , & Gagneux‐Brunon, A. (2020). Intention to participate in a COVID‐19 vaccine clinical trial and to get vaccinated against COVID‐19 in France during the pandemic. Vaccine, 38(45), 7002–7006. 32988688 10.1016/j.vaccine.2020.09.041 PMC7498238

[risa13993-bib-0019] Di Giuseppe, G. , Abbate, R. , Albano, L. , Marinelli, P. , & Angelillo, I. F. (2008). A survey of knowledge, attitudes and practices towards avian influenza in an adult population of Italy. BMC Infectious Diseases, 8(1), 1–8. 18366644 10.1186/1471-2334-8-36 PMC2292195

[risa13993-bib-0020] Dror, A. A. , Eisenbach, N. , Taiber, S. , Morozov, N. G. , Mizrachi, M. , Zigron, A. , Srouji, S. , & Sela, E. (2020). Vaccine hesitancy: The next challenge in the fight against COVID‐19. European Journal of Epidemiology, 35(8), 775–779. 32785815 10.1007/s10654-020-00671-y PMC8851308

[risa13993-bib-0021] Dyer, J. , & Kolic, B. (2020). Public risk perception and emotion on Twitter during the Covid‐19 pandemic. Applied Network Science, 5(1), 1–32. 10.1007/s41109-020-00334-7 PMC7739810 33344760

[risa13993-bib-0022] Eiser, A. R. , & Ellis, G. (2007). Cultural competence and the African American experience with health care: The case for specific content in cross‐cultural education. Academic Medicine, 82(2), 176–183. 17264697 10.1097/ACM.0b013e31802d92ea

[risa13993-bib-0023] Fang, D. , Fang, C.‐L. , Tsai, B.‐K. , Lan, L.‐C. , & Hsu, W.‐S. (2012). Relationships among trust in messages, risk perception, and risk reduction preferences based upon avian influenza in Taiwan. International Journal of Environmental Research and Public Health, 9(8), 2742–2757. 23066394 10.3390/ijerph9082742 PMC3447584

[risa13993-bib-0024] Fisher, K. A. , Bloomstone, S. J. , Walder, J. , Crawford, S. , Fouayzi, H. , & Mazor, K. M. (2020). Attitudes toward a potential SARS‐CoV‐2 vaccine: A survey of US adults. Annals of Internal Medicine, 173(12), 964–973. 32886525 10.7326/M20-3569 PMC7505019

[risa13993-bib-0025] Friis, K. , Lasgaard, M. , Rowlands, G. , Osborne, R. H. , & Maindal, H. T. (2016). Health literacy mediates the relationship between educational attainment and health behavior: A Danish population‐based study. Journal of Health Communication, 21(sup2), 54–60. 27668691 10.1080/10810730.2016.1201175

[risa13993-bib-0026] Gini, C. (1936). On the measure of concentration with special reference to income and statistics. Colorado College Publication, General Series, 208(1), 73–79.

[risa13993-bib-0027] Graffigna, G. , Palamenghi, L. , Boccia, S. , & Barello, S. (2020). Relationship between citizens’ health engagement and intention to take the COVID‐19 vaccine in Italy: A mediation analysis. Vaccines, 8(4), 576–576. 10.3390/vaccines8040576 PMC7711984 33019663

[risa13993-bib-0028] Gupta, G. R. , Parkhurst, J. O. , Ogden, J. A. , Aggleton, P. , & Mahal, A. (2008). Structural approaches to HIV prevention. The Lancet, 372(9640), 764–775. 10.1016/S0140-6736(08)60887-9 18687460

[risa13993-bib-0029] Han, X. , Wang, J. , Zhang, M. , & Wang, X. (2020). Using social media to mine and analyze public opinion related to COVID‐19 in China. International Journal of Environmental Research and Public Health, 17(8), 2788. 10.3390/ijerph17082788 PMC7215577 32316647

[risa13993-bib-0030] Hawkins, D. (2020). Differential occupational risk for COVID‐19 and other infection exposure according to race and ethnicity. American Journal of Industrial Medicine, 63(9), 817–820. 10.1002/ajim.23145 32539166 PMC7323065

[risa13993-bib-0031] He, S. , Chen, S. , Kong, L. , & Liu, W. (2021). Analysis of risk perceptions and related factors concerning COVID‐19 epidemic in Chongqing, China. Journal of Community Health, 46(2), 278–285. 32592160 10.1007/s10900-020-00870-4 PMC7318903

[risa13993-bib-0032] Huang, X. , Li, Z. , Jiang, Y. , Li, X. , & Porter, D. (2020). Twitter reveals human mobility dynamics during the COVID‐19 pandemic. PLoS One, 15(11), e0241957. 33170889 10.1371/journal.pone.0241957 PMC7654838

[risa13993-bib-0033] Hussain, A. , Tahir, A. , Hussain, Z. , Sheikh, Z. , Gogate, M. , Dashtipour, K. , Ali, A. , & Sheikh, A. (2021). Artificial intelligence‐enabled analysis of public attitudes on Facebook and Twitter toward COVID‐19 vaccines in the United Kingdom and the United States: Observational study. Journal of Medical Internet Research, 23(4), e26627. 33724919 10.2196/26627 PMC8023383

[risa13993-bib-0047] Inbar, L. , & Shinan‐Altman, S. (2021). Emotional reactions and subjective health status during the COVID‐19 pandemic in Israel: The mediating role of perceived susceptibility. Psychology, Health & Medicine, 26(1), 75–84. 10.1080/13548506.2020.1858490 33315513

[risa13993-bib-0034] Ingram, D. D. , & Franco, S. J. (2014). 2013 NCHS urban‐rural classification scheme for counties. National Center for Health Statistics, Vital Health Stat 2 (166). 24776070

[risa13993-bib-0035] Iuliano, A. D. , Brunkard, J. M. , Boehmer, T. K. , Peterson, E. , Adjei, S. , Binder, A. M. , Cobb, S. , Graff, P. , Hidalgo, P. , Panaggio, M. J. , Rainey, J. J. , Rao, P. , Soetebier, K. , Wacaster, S. , Ai, C. , Gupta, V. , Molinari, N.‐A. M. , & Ritchey, M. D. (2020). Trends in disease severity and health care utilization during the early omicron variant period compared with previous SARS‐CoV‐2 high transmission periods — United States, December 2020–January 2022. MMWR. Morbidity and Mortality Weekly Report, 71, 146–152. 10.15585/mmwr.mm7104e4 PMC9351529 35085225

[risa13993-bib-0036] Jahangiry, L. , Bakhtari, F. , Sohrabi, Z. , Reihani, P. , Samei, S. , Ponnet, K. , & Montazeri, A. (2020). Risk perception related to COVID‐19 among the Iranian general population: An application of the extended parallel process model. BMC Public Health, 20(1), 1571. 10.1186/s12889-020-09681-7 33076875 PMC7570396

[risa13993-bib-0037] Jang, W. M. , Kim, U.‐N. , Jang, D. H. , Jung, H. , Cho, S. , Eun, S. J. , & Lee, J. Y. (2020). Influence of trust on two different risk perceptions as an affective and cognitive dimension during Middle East respiratory syndrome coronavirus (MERS‐CoV) outbreak in South Korea: Serial cross‐sectional surveys. BMJ Open, 10(3), e033026. 10.1136/bmjopen-2019-033026 PMC7059523 32139484

[risa13993-bib-0038] Janz, N. K. , & Becker, M. H. (1984). The health belief model: A decade later. Health Education Quarterly, 11(1), 1–47. 6392204 10.1177/109019818401100101

[risa13993-bib-0039] Jiang, Y. , Li, Z. , & Ye, X. (2019). Understanding demographic and socioeconomic biases of geotagged Twitter users at the county level. Cartography and Geographic Information Science, 46(3), 228–242.

[risa13993-bib-0040] Johns Hopkins University and Medicine . (2022). COVID‐19 Dashboard by the Center for Systems Science and Engineering (CSSE) at Johns Hopkins University (JHU). https://coronavirus.jhu.edu/map.html

[risa13993-bib-0041] Kim, S. , & Kim, S. (2018). Exploring the determinants of perceived risk of Middle East Respiratory Syndrome (MERS) in Korea. International Journal of Environmental Research and Public Health, 15(6), 1168. 10.3390/ijerph15061168 PMC6025578 29867054

[risa13993-bib-0042] Krikorian, R. V. (Platform Engineering, Twitter Inc.), (Producer). (2013). New Tweets per second record, and how! *Twitter Official Blog*.

[risa13993-bib-0043] Krouse, H. J. (2020). COVID‐19 and the widening gap in health inequity. Otolaryngology— Head and Neck Surgery, 163(1), 65–66. 10.1177/0194599820926463 32366172

[risa13993-bib-0044] Kuper‐Smith, B. J. , Doppelhofer, L. M. , Oganian, Y. , Rosenblau, G. , & Korn, C. (2020). Optimistic beliefs about the personal impact of COVID‐19. *PsyArXiv Preprints, 10* .

[risa13993-bib-0045] Kurten, S. , & Beullens, K. (2021). #Coronavirus: Monitoring the Belgian Twitter discourse on the severe acute respiratory syndrome Coronavirus 2 pandemic. Cyberpsychology, Behavior, and Social Networking, 24(2), 117–122. 32857607 10.1089/cyber.2020.0341

[risa13993-bib-0046] Lestari, P. , & Ulfiana, E. (2021). Beliefs and the correlation with protection health behaviors Covid‐19: A systematic review. Jurnal Keperawatan, 13(3), 605–614.

[risa13993-bib-0048] Loewenstein, G. F. , Weber, E. U. , Hsee, C. K. , & Welch, N. (2001). Risk as feelings. Psychological Bulletin, 127(2), 267–286. 11316014 10.1037/0033-2909.127.2.267

[risa13993-bib-0049] Loomba, R. S. , Aggarwal, G. , Aggarwal, S. , Flores, S. , Villarreal, E. G. , Farias, J. S. , & Lavie, C. J. (2021). Disparities in case frequency and mortality of coronavirus disease 2019 (COVID‐19) among various states in the United States. Annals of Medicine, 53(1), 151–159. 33138653 10.1080/07853890.2020.1840620 PMC7877922

[risa13993-bib-0050] Lyu, J. C. , Le Han, E. , & Luli, G. K. (2021). COVID‐19 vaccine–related discussion on Twitter: Topic modeling and sentiment analysis. Journal of Medical Internet Research, 23(6), e24435. 34115608 10.2196/24435 PMC8244724

[risa13993-bib-0051] Martin, Y. , Cutter, S. L. , Li, Z. , Emrich, C. T. , & Mitchell, J. T. (2020). Using geotagged tweets to track population movements to and from Puerto Rico after Hurricane Maria. Population and Environment, 42(1), 4–27.

[risa13993-bib-0052] Masten, A. S. , & Motti‐Stefandi, F. (2020). Multisystem resilience for children and youth in disaster: Reflections in the context of COVID‐19. Adversity and Resilience Science, 1, 95–106. 32838305 10.1007/s42844-020-00010-w PMC7314620

[risa13993-bib-0053] Millett, G. A. , Jones, A. T. , Benkeser, D. , Baral, S. , Mercer, L. , Beyrer, C. , Honermann, B. , Lankiewicz, E. , Mena, L. , Crowley, J. S. , Sherwood, J. , & Sullivan, P. S. (2020). Assessing differential impacts of COVID‐19 on black communities. Annals of Epidemiology, 47, 37–44. 10.1016/j.annepidem.2020.05.003 32419766 PMC7224670

[risa13993-bib-0054] Min, C. , Shen, F. , Yu, W. , & Chu, Y. (2020). The relationship between government trust and preventive behaviors during the COVID‐19 pandemic in China: Exploring the roles of knowledge and negative emotion. Preventive Medicine, 141, 106288. 33091414 10.1016/j.ypmed.2020.106288 PMC7571476

[risa13993-bib-0055] Moore, J. X. , Langston, M. E. , George, V. , & Coughlin, S. S. (2020). Epidemiology of the 2020 pandemic of COVID‐19 in the state of Georgia: Inadequate critical care resources and impact after 7 weeks of community spread. Journal of the American College of Emergency Physicians Open, 1(4), 527–532. 10.1002/emp2.12127 32838368 PMC7272925

[risa13993-bib-0056] New York Times . (2020). Coronavirus (COVID‐19) data in the United States. https://github.com/nytimes/covid‐19‐data

[risa13993-bib-0057] Odlum, M. , Cho, H. , Broadwell, P. , Davis, N. , Patrao, M. , Schauer, D. , Bales, M. E. , Alcantara, C. , & Yoon, S. (2020). Application of topic modeling to tweets as the foundation for health disparity research for COVID‐19. Studies in Health Technology and Informatics, 272, 24–27. 10.3233/SHTI200484 32604591 PMC7728402

[risa13993-bib-0058] Oh, S.‐H. , Paek, H.‐J. , & Hove, T. (2015). Cognitive and emotional dimensions of perceived risk characteristics, genre‐specific media effects, and risk perceptions: The case of H1N1 influenza in South Korea. Asian Journal of Communication, 25(1), 14–32.

[risa13993-bib-0059] Okonkwo, N. E. , Aguwa, U. T. , Jang, M. , Barré, I. A. , Page, K. R. , Sullivan, P. S. , Beyrer, C. , & Baral, S. (2020). COVID‐19 and the US response: Accelerating health inequities. BMJ Evidence‐Based Medicine, 26(4), 176–179. 10.1136/bmjebm-2020-111426 PMC7299650 32493833

[risa13993-bib-0060] Paakkari, L. , & Okan, O. (2020). COVID‐19: Health literacy is an underestimated problem. Lancet Public Health, 5(5), e249–e250. 32302535 10.1016/S2468-2667(20)30086-4 PMC7156243

[risa13993-bib-0061] Paek, H.‐J. , & Hove, T. (2017). Risk perceptions and risk characteristics. In Oxford research encyclopedia of communication. Oxford University Press.

[risa13993-bib-0062] Perrin, A. , & Anderson, M. (2019). Share of US adults using social media, including Facebook, is mostly unchanged since 2018. https://www.pewresearch.org/fact-tank/2019/04/10/share-of-us-adults-using-social-media-including-facebook-is-mostly-unchanged-since-2018

[risa13993-bib-0063] Prati, G. , Pietrantoni, L. , & Zani, B. (2011). Compliance with recommendations for pandemic influenza H1N1 2009: The role of trust and personal beliefs. Health Education Research, 26(5), 761–769. 21613380 10.1093/her/cyr035

[risa13993-bib-0064] Rimal, R. N. , & Real, K. (2003). Perceived risk and efficacy beliefs as motivators of change: Use of the risk perception attitude (RPA) framework to understand health behaviors. Human Communication Research, 29(3), 370–399.

[risa13993-bib-0065] Rosenberg, H. , Syed, S. , & Rezaie, S. (2020). The Twitter pandemic: The critical role of Twitter in the dissemination of medical information and misinformation during the COVID‐19 pandemic. Canadian Journal of Emergency Medicine, 22(4), 418–421. 32248871 10.1017/cem.2020.361 PMC7170811

[risa13993-bib-0066] Rubin, G. J. , Amlôt, R. , Page, L. , & Wessely, S. (2009). Public perceptions, anxiety, and behaviour change in relation to the swine flu outbreak: Cross sectional telephone survey. BMJ, *339*. 10.1136/bmj.b2651 PMC2714687 19574308

[risa13993-bib-0067] Rudisill, C. (2013). How do we handle new health risks? Risk perception, optimism, and behaviors regarding the H1N1 virus. Journal of Risk Research, 16(8), 959–980.

[risa13993-bib-0068] Rui, J. R. , Yang, K. , & Chen, J. (2021). Information sources, risk perception, and efficacy appraisal's prediction of engagement in protective behaviors against COVID‐19 in China: Repeated cross‐sectional survey. JMIR Human Factors, 8(1), e23232. 33338027 10.2196/23232 PMC7806274

[risa13993-bib-0069] Rypdal, K. , Bianchi, F. M. , & Rypdal, M. (2020). Intervention fatigue is the primary cause of strong secondary waves in the COVID‐19 pandemic. International Journal of Environmental Research and Public Health, 17(24), 9592. 10.3390/ijerph17249592 PMC7767484 33371489

[risa13993-bib-0070] Savadori, L. , & Lauriola, M. (2021). Risk perception and protective behaviors during the rise of the COVID‐19 outbreak in Italy. Frontiers in Psychology, 11, 3822. 10.3389/fpsyg.2020.577331 PMC7838090 33519593

[risa13993-bib-0071] Sherman, S. M. , Smith, L. E. , Sim, J. , Amlôt, R. , Cutts, M. , Dasch, H. , Rubin, G. J. , & Sevdalis, N. (2021). COVID‐19 vaccination intention in the UK: Results from the COVID‐19 vaccination acceptability study (CoVAccS), a nationally representative cross‐sectional survey. Human Vaccines & Immunotherapeutics, 17(6), 1612–1621. 33242386 10.1080/21645515.2020.1846397 PMC8115754

[risa13993-bib-0072] Shmueli, L. (2021). Predicting intention to receive COVID‐19 vaccine among the general population using the health belief model and the theory of planned behavior model. BMC Public Health, 21(1), 1–13. 33902501 10.1186/s12889-021-10816-7 PMC8075011

[risa13993-bib-0073] Slovic, P. (1987). Perception of risk. Science, 236(4799), 280–285. 3563507 10.1126/science.3563507

[risa13993-bib-0074] Slovic, P. , Finucane, M. L. , Peters, E. , & MacGregor, D. G. (2007). The affect heuristic. European Journal of Operational Research, 177(3), 1333–1352.

[risa13993-bib-0075] Slovic, P. , Fischhoff, B. , & Lichtenstein, S. (1982). Why study risk perception? Risk Analysis, 2(2), 83–93.

[risa13993-bib-0076] Sobkow, A. , Zaleskiewicz, T. , Petrova, D. , Garcia‐Retamero, R. , & Traczyk, J. (2020). Worry, risk perception, and controllability predict intentions toward COVID‐19 preventive behaviors. Frontiers in Psychology, 11, 582720. 33329239 10.3389/fpsyg.2020.582720 PMC7710521

[risa13993-bib-0077] Strathdee, S. A. , Nobles, A. L. , & Ayers, J. W. (2019). Harnessing digital data and data science to achieve 90‐90‐90 goals to end the HIV epidemic. Current Opinion in HIV and AIDS, 14(6), 481–485. 31449089 10.1097/COH.0000000000000584 PMC6956609

[risa13993-bib-0078] Tagini, S. , Brugnera, A. , Ferrucci, R. , Mazzocco, K. , Compare, A. , Silani, V. , Pravettoni, G. , & Poletti, B. (2021). It won't happen to me! Psychosocial factors influencing risk perception for respiratory infectious diseases: A scoping review. Applied Psychology: Health and Well‐Being, 13(4), 835–852. 33855817 10.1111/aphw.12274 PMC8250503

[risa13993-bib-0079] Tan, S. B. , deSouza, P. , & Raifman, M. (2021). Structural Racism and COVID‐19 in the USA: A county‐level empirical analysis. Journal of Racial and Ethnic Health Disparities, 9, 236–246. 10.1007/s40615-020-00948-8 33469868 PMC7815192

[risa13993-bib-0080] Tausczik, Y. R. , & Pennebaker, J. W. (2010). The psychological meaning of words: LIWC and computerized text analysis methods. Journal of Language and Social Psychology, 29(1), 24–54.

[risa13993-bib-0081] Tracker, T. C. R. D. (2020). COVID‐19 is affecting Black, Indigenous, Latinx, and other people of color the most. https://covidtracking.com/race

[risa13993-bib-0082] Tsoy, D. , Tirasawasdichai, T. , & Kurpayanidi, K. I. (2021). Role of social media in shaping public risk perception during COVID‐19 pandemic: A theoretical review. International Journal of Management Science and Business Administration, 7(2), 35–41.

[risa13993-bib-0083] Twitter . (2021). https://developer.twitter.com/en/docs/tutorials/tweet‐geo‐metadata

[risa13993-bib-0084] Twitter . (2022). Volume streams. https://developer.twitter.com/en/docs/twitter‐api/tweets/volume‐streams/introduction

[risa13993-bib-0085] U.S. Census . (2019). American Community Survey 5‐Year Data (2009–2018). https://www.census.gov/data/developers/data‐sets/acs‐5year.2018.html

[risa13993-bib-0086] van der Weerd, W. , Timmermans, D. R. , Beaujean, D. J. , Oudhoff, J. , & van Steenbergen, J. E. (2011). Monitoring the level of government trust, risk perception and intention of the general public to adopt protective measures during the influenza A (H1N1) pandemic in the Netherlands. BMC Public Health, 11(1), 1–12. 21771296 10.1186/1471-2458-11-575 PMC3152536

[risa13993-bib-0087] Visschers, V. H. , & Siegrist, M. (2018). Differences in risk perception between hazards and between individuals. In Psychological perspectives on risk and risk analysis (pp. 63–80). Springer.

[risa13993-bib-0088] von Gottberg, C. , Krumm, S. , Porzsolt, F. , & Kilian, R. (2016). The analysis of factors affecting municipal employees’ willingness to report to work during an influenza pandemic by means of the extended parallel process model (EPPM). BMC Public Health, 16(1), 1–12. 26757713 10.1186/s12889-015-2663-8 PMC4711035

[risa13993-bib-0089] Wang, F. , Wei, J. , Huang, S.‐K. , Lindell, M. K. , Ge, Y. , & Wei, H.‐L. (2018). Public reactions to the 2013 Chinese H7N9 Influenza outbreak: Perceptions of risk, stakeholders, and protective actions. Journal of Risk Research, 21(7), 809–833.

[risa13993-bib-0090] Weber, E. U. (2017). Understanding public risk perception and responses to changes in perceived risk. In Policy shock: Recalibrating risk and regulation after oil spills, nuclear accidents and financial crises, (pp. 82–106). Cambridge University Press.

[risa13993-bib-0091] WHO Regional Office for Europe . (2020). Pandemic fatigue – Reinvigorating the public to prevent COVID‐19. Policy framework for supporting pandemic prevention and management. https://apps.who.int/iris/bitstream/handle/10665/335820/WHO‐EURO‐2020‐1160‐40906‐55390‐eng.pdf

[risa13993-bib-0092] Wise, T. , Zbozinek, T. D. , Michelini, G. , Hagan, C. C. , & Mobbs, D. (2020). Changes in risk perception and self‐reported protective behaviour during the first week of the COVID‐19 pandemic in the United States. Royal Society Open Science, 7(9), 200742. 10.1098/rsos.200742 33047037 PMC7540790

[risa13993-bib-0093] Young, S. D. , Rivers, C. , & Lewis, B. (2014). Methods of using real‐time social media technologies for detection and remote monitoring of HIV outcomes. Preventive Medicine, 63, 112–115. 24513169 10.1016/j.ypmed.2014.01.024 PMC4031268

[risa13993-bib-0094] Young, S. D. , & Zhang, Q. (2018). Using search engine big data for predicting new HIV diagnoses. PLoS One, 13(7), e0199527. 30001360 10.1371/journal.pone.0199527 PMC6042696

[risa13993-bib-0095] Yousefinaghani, S. , Dara, R. , Mubareka, S. , Papadopoulos, A. , & Sharif, S. (2021). An analysis of COVID‐19 vaccine sentiments and opinions on Twitter. International Journal of Infectious Diseases, 108, 256–262. 34052407 10.1016/j.ijid.2021.05.059 PMC8157498

[risa13993-bib-0096] Zeballos Rivas, D. R. , Lopez Jaldin, M. L. , Nina Canaviri, B. , Portugal Escalante, L. F. , Alanes Fernández, A. M. , & Aguilar Ticona, J. P. (2021). Social media exposure, risk perception, preventive behaviors and attitudes during the COVID‐19 epidemic in La Paz, Bolivia: A cross sectional study. PLoS One, 16(1), e0245859. 33481945 10.1371/journal.pone.0245859 PMC7822287

[risa13993-bib-0097] Zeng, Q. T. , & Tse, T. (2006). Exploring and developing consumer health vocabularies. Journal of the American Medical Informatics Association, 13(1), 24–29. 16221948 10.1197/jamia.M1761 PMC1380193

[risa13993-bib-0098] Zephyrin, L. , Radley, D. C. , Getachew, Y. , Baumgartner, J. C. , & Schneider, E. C. (2020). COVID‐19 more prevalent, deadlier in U.S. counties with higher Black populations. https://www.commonwealthfund.org/blog/2020/covid‐19‐more‐prevalent‐deadlier‐us‐counties‐higher‐black‐populations

[risa13993-bib-0099] Zhang, C. H. , & Schwartz, G. G. (2020). Spatial disparities in coronavirus incidence and mortality in the United States: An ecological analysis as of May 2020. Journal of Rural Health, 36(3), 433–445. 10.1111/jrh.12476 PMC7323165 32543763

[risa13993-bib-0100] Zhu, X. , Kim, Y. , & Park, H. (2020). Do messages spread widely also diffuse fast? Examining the effects of message characteristics on information diffusion. Computers in Human Behavior, 103, 37–47.

