# Supplementary material for: Three dimensions of COVID‐19 risk perceptions and their socioeconomic correlates in the United States: A social media analysis
Source: Risk Anal. 2022 Jul 13:10.1111/risa.13993. doi: 10.1111/risa.13993 (PMC9350290; doi:10.1111/risa.13993)

**Appendix Table 1. Twitter sources that indicate non-human-posted tweets selected by manual checking**

Echofon, Endomondo, Fenix for Android, Flamingo for Android, Foursquare, Gay Los Angeles, Gay Santa Monica,Gay West Hollywood, Hootsuite, Instagram, iOS,OSX,PlumeforAndroid,SoundHound,Squarespace,Talon (Plus),Talon Android, Talon Plus, Tweet It! for Windows, Tweetbot for iS, Tweetbot for Mac, TweetCaster for Android, TweetCaster for iOS, Tweetings for iPad, Tweetings for Android, Tweetings for Android Holo, Tweetings for Android Tablets, Tweetings for iPhone, Tweetlogix, twicca, Twidere for Android #4,Twidere for Android #5,Twidere for Android #7,Twishort Client, Twittelator, Twitter Dashboard for iPhone, Twitter Engage for iPhone, Twitter for Android, Twitter for iPhone, Twitter for Android, Twitter for Android Tablets, Twitter for Apple Watch, Twitter for BlackBerry, Twitter for Calendar, Twitter for iPad, Twitter for iPhone, Twitter for Mac, Twitter for Windows, Twitter for Windows Phone, Untappd, Tweetbot for iOS, Foursquare Swarm

**Appendix Table 2. Key events regarding COVID-19 pandemic from January 2020 to December 2021**

| **Date** | **Events** |
| --- | --- |
| 1/3/2020 | China officially notifies the WHO of an outbreak. |
| 1/20/2020 | United States confirms its first case in Washington state, a man who traveled to the Wuhan area. |
| 1/27/2020 | United States extends screening to twenty airports. |
| 1/30/2020 | The WHO declares the coronavirus outbreak as a Public Health Emergency of International Concern (PHEIC). |
| 2/4/2020 | The U.S. Food and Drug Administration issues an emergency use authorization for CDC’s diagnostic test. |
| 2/11/2020 | WHO names the disease COVID-19, short for “coronavirus disease 2019.” |
| 2/24/2020 | The Trump administration sends a budget request to Congress for $2.5 billion to fight COVID-19. |
|  | The U.S. stock market plummeted over coronavirus fears, after the Dow Jones Industrial Average experienced the worst day in two years. |
| 2/25/2020 | San Francisco becomes the first U.S. city to declare a state of emergency over COVID-19. |
| 2/26/2020 | President Donald Trump names Vice President Mike Pence to lead the U.S. coronavirus response. |
| 2/27/2020 | U.S. CDC widens its testing guidelines |
| 2/29/2020 | The United States reports its first death, a man in his fifties with an underlying health condition. |
| 3/4/2020 | House passes $8.3 billion emergency coronavirus bill |
| 3/6/2020 | CDC urges those over 60 to stay indoors |
| 3/10/2020 | Michigan, Vermont, North Carolina, Massachusetts, and Colorado all declare a state of emergency (23 states total). |
| 3/11/2020 | WHO declares the coronavirus outbreak a pandemic |
|  | United States announces level 3 travel advisory and suspended entry to all foreign nationals traveling from China, Iran, and certain European countries at any point during the 14 days prior to their scheduled travel to the U.S. |
|  | NBA suspends their season |
| 3/12/2020 | U.S. stocks record their worst day since 1987 |
| 3/13/2020 | Donald Trump declares a state of National Emergency under Stafford Act |
|  | Sixteen states including: Alabama, Wisconsin, Maryland, Ohio, Kentucky, Washington, Wisconsin,  West Virginia, Illinois, Pennsylvania, Louisiana, Rhode Island, Michigan, New Mexico, Oregon, Virginia, Utah have announced school closures |
|  | WHO declares Europe the new epicenter of the outbreak |
| 3/14/2020 | The United States reaches 2,750 Coronavirus cases. All states have reported cases except West Virginia. |
| 3/15/2020 | New York City public schools close. |
|  | New York Mayor Bill DeBlasio signs an executive order stating that New York City hospitals are required to cancel elective surgeries. |
|  | Massachusetts bans eating and drinking at bars until at least April 17. |
|  | 29 States announce school closures. |
| 3/16/2020 | CDC reports over 4,000 coronavirus cases in the United States. |
| 3/17/2020 | West Virginia confirms its first coronavirus case—making the virus present in all 50 United States. |
| 3/18/2020 | Trump signs the Families First Coronavirus Response Act into law . |
|  | The United States and Canada suspend non-essential travel between the two countries. |
| 3/19/2020 | California issues a stay-at-home order for all of its 40 million residents. |
|  | Vermont reports first coronavirus-related deaths |
|  | The United States CDC reports more than 13,000 coronavirus cases. |
|  | U.S. Department of States issues a level-four “Do Not Travel” advisory. |
| 3/20/2020 | U.S. stocks close their worst week since the 2008 financial crisis. |
|  | Trump invokes Defense Production Act to disperse medical supplies to hospitals. |
|  | The United States CDC reports more than 18,000 coronavirus cases. |
| 3/23/2020 | WHO announces the “pandemic is accelerating.” |
| 3/24/2020 | U.S. National Guard is activated in all 50 States. |
|  | United States reaches 50,000 coronavirus cases. |
| 3/26/2020 | New York City becomes the epicenter of the US outbreak. Reported coronavirus cases double every three days |
|  | United States death toll reaches one thousand |
| 3/27/2020 | UK Prime Minister Boris Johnson tests positive for coronavirus. |
| 3/29/2020 | President Trump extends social distancing guidelines until April 30. |
|  | The United States passes 140,000 coronavirus cases—more than any other country in the world. |
| 3/30/2020 | President Trump announces more than 1 million Americans have been tested for coronavirus. |
| 3/31/2020 | The United States unveils a model which project that 100,000 Americans could die from the coronavirus. |
| 4/2/2020 | 6.6 million Americans file for unemployment over the course of the last week, bringing the total number of unemployment claims to over 10 million. |
|  | Almost 91 percent of Americans are ordered to stay at home |
|  | The White House encourages all Americans to wear masks in public. |
| 4/3/2020 | The United States confirms 32,000 new cases in one day, setting a new record for the largest jump in daily cases |
|  | U.S. President Donald Trump invokes the Defense Productions Act to halt the export of masks and other personal protection equipment. |
| 4/5/2020 | The United States reports 1,300 coronavirus deaths in one day, its highest daily spike. |
|  | The CDC begins testing blood for coronavirus immunity |
| 4/6/2020 | United States death toll passes 10,000 |
| 4/9/2020 | 6.6 million Americans file for unemployment claims in the past week, bringing the total number of unemployment claims filed in the past three weeks to over 17 million. |
| 4/10/2020 | New York City reports more coronavirus cases than any country. |
| 4/11/2020 | The United States surpasses Italy for having the most confirmed coronavirus deaths in the world. |
| 4/16/2020 | President Donald J. Trump unveils a set of guidelines for opening up America giving liberty to state Governors to choose whether they want lift restrictions statewide or on a county-by-county basis. |
|  | An additional 5.2 million Americans file for unemployment over the past week, bringing the total number to 22 million Americans filing since President Donald J. Trump declared a state of emergency four weeks ago. This job loss is comparable to Great Depression statistics. |
| 4/17/2020 | U.S. Vice President, Mike Pence declares that the country has enough tests for a phase one reopening. |
|  | Texas Governor, Greg Abbott, makes an executive decision to begin reopening Texas beginning in May. This decision makes Texas one of the first states to loosen restrictions. |
| 4/20/2020 | President Donald J. Trump announces he will temporarily suspend immigration to the United States for 60 days by executive order. |
| 4/21/2020 | President Donald J. Trump effectively suspends immigration to the United States. Green card recipients will be blocked from moving to the country but temporary workers on nonimmigrant visas will be allowed in. |
| 4/23/2020 | The White House announces it has enough test kits for a phase one reopening. |
| 4/24/2020 | The United States’ coronavirus death toll passes 50,000. |
| 4/25/2020 | Over a quarter of the world’s coronavirus deaths are in the United States. |
| 4/28/2020 | The United States records over one million coronavirus cases |
| 5/5/2020 | Trump administration considers phasing out coronavirus task force. |
| 5/27/2020 | Coronavirus deaths in the United States pass 100,000. |
| 6/8/2020 | New York City begins phase I reopening |
| 6/9/2020 | Since Memorial Day in the United States, nine states report increase in hospitalizations due to coronavirus. |
| 6/11/2020 | As the United States exceeds 2 million confirmed coronavirus cases, new model suggests spikes in death toll in September and later months. |
| 6/15/2020 | The United States surpasses 115,000 coronavirus deaths as new cases increase across more than 12 states |
| 6/20/2020 | Florida and South Carolina report sharp spikes in new cases, breaking single-day records for third day in a row |
| 6/22/2020 | Citing pandemic concerns, President Trump issues restrictions on immigration  to the United States, suspending most H1-B, H2-B, and H-4 visas. |
| 6/23/2020 | FDA warns against the use of hand sanitizers containing methanol, a toxic substance. |
| 6/24/2020 | The United States reports its highest daily total of new coronavirus cases. |
|  | Twenty-six U.S. states see a rise in coronavirus cases since easing up on lockdown restrictions. |
| 6/25/2020 | U.S. CDC estimates that more than 20 million individuals may have had coronavirus in the United States thus far. |
| 6/26/2020 | The United States sees its highest daily increase in confirmed coronavirus cases. |
| 6/27/2020 | Twelve U.S. states slow reopening measures as new cases increase across the country. |
| 6/30/2020 | The United States acquires over 500,000 doses of remdesivir from Gilead, all of its production for the month of July and 90 percent of August and September. |
| 7/1/2020 | Texas governor, Greg Abbott, mandates face masks in public |
| 7/2/2020 | Florida reports over 10,000 new coronavirus cases, marking a new single-day record for the state. |
| 7/3/2020 | The United States reports over 55,000 new coronavirus cases, marking a new daily global record. |
| 7/6/2020 | California orders six additional counties to close indoor operations for restaurants, bars, and other businesses as coronavirus cases increase in the state |
| 7/7/2020 | President Donald Trump formally notifies Congress and the United Nations of U.S. withdrawal from WHO |
| 7/8/2020 | Intensive care units in hospitals across Florida and Arizona reach full capacity due to surges in coronavirus cases |
| 7/9/2020 | Single-day records are reported across four states in the United States. |
| 7/10/2020 | The United States reports 63,247 new coronavirus cases, its highest single-day increase to date. |
| 7/11/2020 | Louisiana mandates masks statewide amid rising cases and hospitalizations. |
| 7/12/2020 | President Donald Trump makes his first public appearance wearing a face-mask. |
|  | Florida marks 15,300 new cases, the highest single-day increase of any U.S. state since the pandemic began. |
| 7/13/2020 | New York City reports no new coronavirus deaths in a 24-hour period. |
|  | California's two largest public school districts, Los Angeles and San Diego, announce they will be online-only for the fall semester. |
|  | WHO reports that the U.S. and Brazil made up half of the daily increase in coronavirus cases globally. |
| 7/14/2020 | Moderna, the first potential coronavirus vaccine tested in humans, is demonstrated to show a positive immune response. |
|  | The White House orders hospitals to bypass the CDC and send COVID-19 case data directly to Washington. |
| 7/15/2020 | Single-day tallies for cases, hospitalizations, and deaths reach highs across several states including Florida, Oklahoma, and Mississippi. |
|  | Walmart, the world's largest retailer, requires all shoppers to wear face-masks in all 5,000 of its U.S. stores. |
| 7/16/2020 | Georgia's governor issues an executive order rescinding mask mandates made by local governments across the state. |
| 7/18/2020 | The FDA issues its first emergency use authorization for COVID-19 pool testing. |
| 7/21/2020 | The United States accuses China of hacking COVID-19 vaccine research trials. |
| 7/22/2020 | Washington, D.C. issues a new mask mandate as cases rise. |
|  | The United States charges two Chinese hackers accused of targeting institutions working on vaccine research in the U.S., Germany, UK, and Japan, among other nations. |
| 7/25/2020 | COVID-19 hospitalizations are up 79 percent in three weeks in Florida. |
| 7/27/2020 | Phase III clinical trials for a COVID-19 vaccine, developed by Moderna, begin in the United States. |
| 7/29/2020 | Russia reports its on-track to approve the first COVID-19 vaccine in mid-August. |
| 8/1/2020 | Mississippi has the highest COVID-19 positivity rate in the United States. |
| 8/7/2020 | New York Governor, Andrew Cuomo announces New York City schools can reopen in the fall if COVID-19 cases remain low. |
| 8/10/2020 | Moderna and the Trump administration negotiate a deal to supply the United States with 100 million doses of its experimental COVID-19 vaccine. |
| 8/13/2020 | U.S. presidential candidate Joe Biden calls for a three-months national mask mandate. |
| 8/17/2020 | New York Governor, Andrew Cuomo announces COVID-19 infection rate below one percent for the tenth consecutive day. |
| 8/19/2020 | The Trump Administration's Operation Warp Speed announces a COVID-19 vaccine will likely be made available next spring. |
| 8/23/2020 | The FDA issues an emergency authorization for convalescent plasma treatment against COVID-19. |
| 8/24/2020 | White House officials announce the possibility of fast-tracking a COVID-19 vaccine before phase III trials are completed |
|  | New COVID-19 cases are decreasing in half of U.S. States |
| 8/27/2020 | The White House announces the purchase of 150 million rapid COVID-19 tests. |
| 8/28/2020 | New York announces its lowest rate of COVID-19 infection since the pandemic began, marking three consecutive weeks of an infection rate below one percent. |
|  | The FDA expands emergency authorization of remdesivir for hospitalized COVID-19 patients. |
| 9/1/2020 | The White House announces it will resume tours at 18 percent capacity following six-month hiatus |
|  | The National Institutes of Health advised doctors to not use convalescent plasma as a COVID-19 treatment until more research is conducted. |
|  | Utah State University issues mandatory quarantine after detecting COVID-19 in water supply. |
| 9/2/2020 | Dr. Moncef Slaoui, the chief adviser for the White House vaccine program, said that a COVID-19 vaccine before November would be “extremely unlikely but not impossible.” |
| 9/4/2020 | Moderna slows down phase III vaccine trials to expand testing to at-risk minorities. |
|  | One study finds that Russia's COVID-19 vaccine generated a "strong immune response." |
| 9/7/2020 | New York's positive test rate stays below one percent for 30 consecutive days |
| 9/9/2020 | The United States announces it will stop screening international arrivals for COVID-19. |
| 9/16/2020 | Director of the Centers for Disease Control and Prevention, Dr. Robert Redfield, announces that a COVID-19 vaccine likely will not be widely available until mid-2021. |
| 9/25/2020 | The United States passes 7 million COVID-19 cases. |
| 9/28/2020 | New York's positive COVID-19 test rate reaches 1.5 percent, reflecting a national increase in cases. |
| 9/29/2020 | Moderna's COVID-19 vaccine shows acceptable safety |
|  | New York City's COVID-19 positivity rate reaches 3.25 percent-- its highest figure since June. |
| 9/30/2020 | The CDC announces a Cruise Ship No Sail Order, effective through October 31. |
| 10/1/2020 | Advisor to U.S. President, Hope Hicks, tests positive for COVID-19. |
|  | New York City's hotspots in Queens and Brooklyn, reach a 6.5 COVID-19 positivity rate. |
| 10/2/2020 | U.S. President, Donald J. Trump, and First Lady, Melania Trump, test positive for COVID-19. |
|  | President Donald J. Trump is flown to Walter Reed hospital to receive treatment for the coronavirus. |
| 10/5/2020 | President Donald J. Trump returns to the White House from Walter Reed hospital. |
| 10/8/2020 | Top White House aid, Stephen Miller, tests positive for COVID-19. |
|  | White House COVID-19 outbreak reaches at least 34 people |
| 10/12/2020 | Johnson & Johnson pauses its COVID-19 vaccine trails after a participant contracts an unexplained illness. |
| 10/16/2020 | U.S. passed 8 million COVID-19 cases. |
| 10/20/2020 | The United States reports 58,387 new COVID-19 cases in a 24-hour period, its highest figure since July |
| 10/30/2020 | U.S. passed 9 million COVID-19 cases. |
| 11/4/2020 | US Reports Unprecedented 100,000 New Cases in 1 Day |
| 11/9/2020 | U.S. passed 10 million COVID-19 cases. |
| 11/15/2020 | U.S. passed 11 million COVID-19 cases. |
| 11/21/2020 | U.S. passed 12 million COVID-19 cases, with a record high of more than 200,000 cases being reported in the preceding days. |
| 12/2/2020 | U.S. passed 14 million cases and also set records for the highest number of daily deaths (3,157), new infections (nearly 205k), and hospitalizations (over 100k) |
| 12/3/2020 | The Advisory Committee on Immunization Practices (ACIP) recommends that health care professionals and residents of long-term care facilities be offered COVID-19 vaccine first in the initial phases of the COVID-19 vaccination program. |
| 12/11/2020 | Food and Drug Administration issues an Emergency Use Authorization (EUA) for the first COVID-19 vaccine – the Pfizer-BioNTech COVID-19 vaccine. |
| 12/12/2020 | The Advisory Committee on Immunization Practices (ACIP) issues interim recommendation for the use of the Pfizer-BioNTech COVID-19 vaccine in persons aged 16 years or older for the prevention of COVID-19. |
| 12/14/2020 | United States coronavirus (COVID-19) death toll surpasses 300,000. |
| 12/14/2020 | US officials announce the first doses of the FDA authorized Pfizer vaccine have been delivered to all 50 states, the District of Columbia and Puerto Rico. |
| 12/14/2020 | Sandra Lindsay, a nurse in New York, becomes the first American outside a clinical trial to receive the COVID-19 vaccine. |
| 12/18/2020 | The U.S.Food and Drug Administration issues an Emergency Use Authorization for the second COVID-19 vaccine – the Moderna COVID-19 vaccine. |
| 12/19/2020 | The Advisory Committee on Immunization practices (ACIP) issues an interim recommendation for the use of the Moderna COVID-19 vaccine in persons aged 18 years or older for the prevention of COVID-19. |
| 12/21/2020 | U.S. passed 18 million cases. |
| 12/24/2020 | It is estimated that more than 1 million people in the U.S. are vaccinated against COVID-19. |
| 12/29/2020 | First confirmed case of the new SARS-CoV-2 variant from the United Kingdom was reported in Colorado. |
| 12/30/2020 | A confirmed case of the new SARS-CoV-2 variant from the United Kingdom was reported in California. |
| 1/1/2021 | U.S. passed 20 million cases, representing an increase of more than one million over the past week. |
| 1/18/2021 | U.S COVID-19 death toll surpasses 400,000. |
| 1/20/2021 | One year anniversary of the first reported case of COVID-19 in the U.S. Snohomish County, Washington |
| 1/22/2021 | U.S. passed 25 million cases |
| 1/25/2021 | First U.S. case of Brazil variant of coronavirus reported in Minnesota. |
| 1/28/2021 | First U.S. case of South African variant of coronavirus reported in South Carolina. |
| 2/1/2021 | At home tests to be put into distribution in the U.S. by Australian company Ellume. |
| 2/21/2021 | U.S. COVID-19 death toll surpasses 500,000. |
| 2/27/2021 | FDA approves emergency use authorization for Johnson and Johnson one shot COVID-19 vaccine. |
| 2/28/2021 | Media Statement from CDC Director Rochelle P. Walensky, MD, MPH, on Signing the Advisory Committee on Immunization Practices’ Recommendation to Use Janssen’s COVID-19 Vaccine in People 18 and Older |
| 3/11/2021 | Media Statement from CDC Director Rochelle P. Walensky, MD, MPH, In Observance of One-Year Pandemic Milestone |
| 3/11/2021 | President Joseph Biden announces a federal vaccine website where users can find vaccines near them. He also directs all states, tribes, and territories to make all adults eligible for the vaccine by May 1st. |
| 3/13/2021 | U.S. surpasses 100 million vaccinations administered. |
| 3/24/2021 | U.S. passed 30 million cases |
| 4/2/2021 | CDC announces fully vaccinated individuals can travel safely domestically in the U.S. without a COVID test first. |
| 4/6/2021 | Nearly 80 percent of teachers, school staff, and childcare workers receive at least one shot of COVID-19 vaccine |
| 4/21/2021 | U.S. surpasses 200 million vaccinations administered. |
| 6/1/2021 | The Delta variant, first identified in India in late 2020, becomes the dominant variant in the U.S. The variant kicks off a third wave of infections during the summer of 2021 |
| 6/15/2021 | U.S. passed 600,000 deaths |
| 7/27/2021 | After a substantial upswing in cases due to the Delta variant, CDC releases updated guidance for everyone in areas with substantial or high transmission to wear a mask while indoors. |
| 8/1/2021 | U.S. passed 35 million cases |
| 8/30/2021 | ACIP recommends Pfizer-BioNTech’s vaccine for people ages 16 years and older. |
| 9/7/2021 | U.S. passed 40 million cases. |
| 9/20/2021 | COVID-19 had killed over 675,000 Americans, the estimated number of American deaths from the Spanish flu in 1918. As a result, COVID-19 became the deadliest respiratory pandemic in American history. |
| 10/1/2021 | U.S. passed 700,000 deaths. |
| 10/18/2021 | U.S. passed 45 million cases. |
| 10/21/2021 | CDC endorses ACIP recommendation for COVID-19 booster shots for people who are 65 years and older, and people 18 years and older who live in long-term care settings, have underlying medical conditions, and who live or work in high-risk settings. |
| 11/2/2021 | CDC endorses ACIP recommendation that children ages 5 to 11 years be vaccinated against COVID-19 with the Pfizer-BioNTech pediatric vaccine. |
| 11/19/2021 | CDC expands recommendations for booster shots to include all adults ages 18 years and older who received a Pfizer-BioNTech or Moderna vaccine at least six months after their second dose. |
| 11/29/2021 | CDC recommends that everyone over 18 years old who received a Pfizer or Moderna vaccine receive a COVID-19 booster shot 6 months after they are fully vaccinated. |
| 12/1/2021 | First Confirmed Case of Omicron Variant Detected in the United States |
| 12/9/2021 | CDC Expands COVID-19 Booster Recommendations to 16-and-17-year-olds |
| 12/22/2021 | The FDA authorizes Pfizer's antiviral pill, Paxlovid, to treat Covid-19, the first antiviral Covid-19 pill authorized in the United States for ill people to take at home, before they get sick enough to be hospitalized. |
| 12/27/2021 | CDC shortens the recommended times that people should isolate when they've tested positive for Covid-19 from 10 days to five days if they don't have symptoms -- and if they wear a mask around others for at least five more days. The CDC also shortens the recommended time for people to quarantine if they are exposed to the virus to a similar five days if they are vaccinated. |

**Appendix Table 3. Urban-rural distribution of the selected counties (with Twitter users greater than 100) based on the 2013 National Center for Health Statistics (NCHS)** **Urban-Rural Classification Scheme for Counties**

| **2013 NCHS Urban-Rural Classification** | **Included counties in the study** | | **All the counties in the US** | |
| --- | --- | --- | --- | --- |
|  | **Number of counties** | **Percentage** | **Number of counties** | **Percentage** |
| Large central metro | 68 | 6.59% | 68 | 2.19% |
| Large fringe metro | 249 | 24.13% | 368 | 11.84% |
| Medium metro | 239 | 23.16% | 369 | 11.87% |
| Small metro | 213 | 20.64% | 335 | 10.78% |
| Micropolitan/Noncore | 263 | 25.48% | 1,948 | 62.66% |
| Total | 1,032 | 100% | 3,109 | 100% |

Source: Ingram, D. D., & Franco, S. J. (2014). *2013 NCHS urban-rural classification scheme for counties*: US Department of Health and Human Services, Centers for Disease Control and Prevention.

**Appendix Figure 1 Spatial distribution of the selected counties with more than 100 Twitter users.**


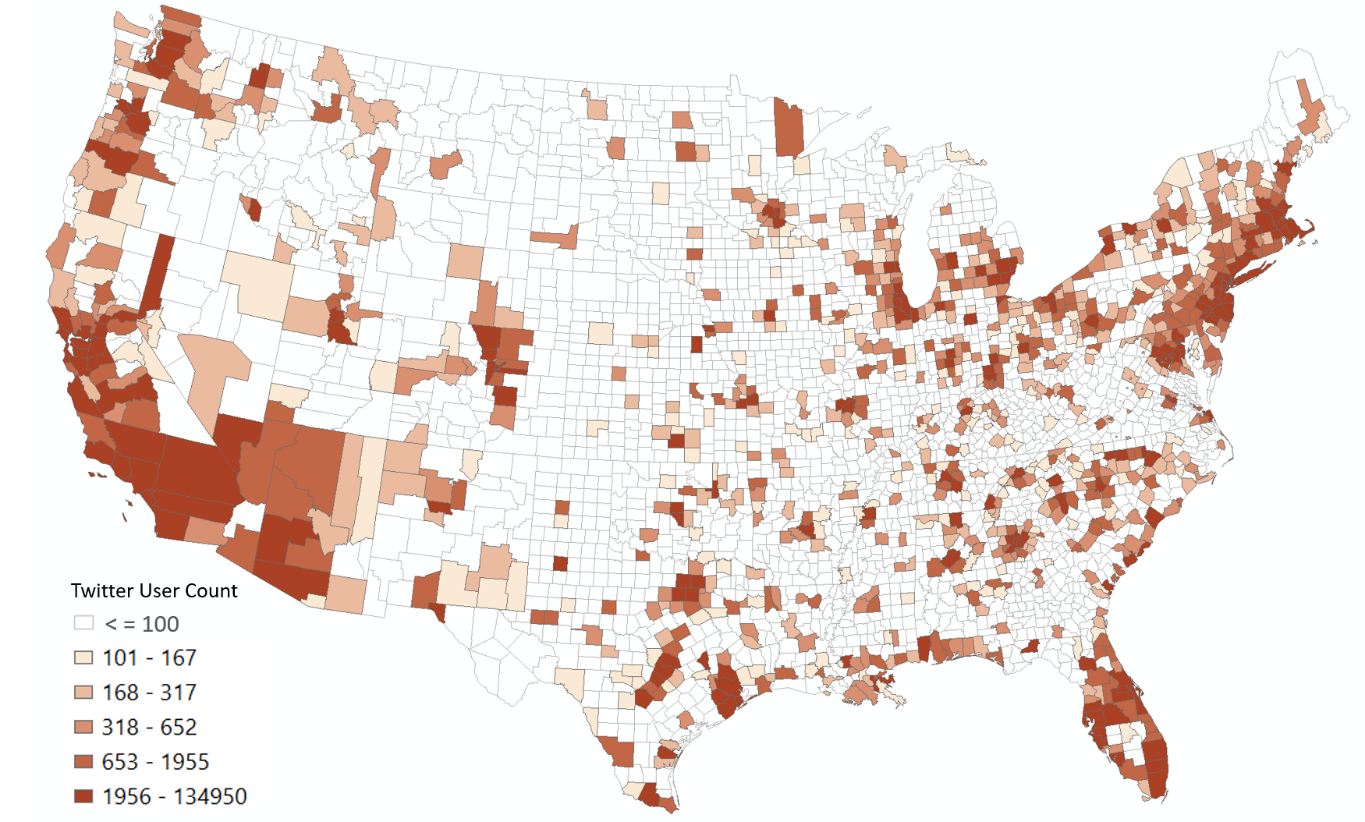

Supplement: Supplementary file 1 — Appendix Table 1. Twitter sources that indicate non‐human‐posted tweets selected by manual checking Appendix Table 2. Key events regarding COVID‐19 pandemic from January 2020 to December 2021 Appendix Table 3. Urban‐rural distribution of the selected counties (with Twitter users greater than 100) based on the 2013 National Center for Health Statistics (NCHS) Urban‐Rural Classification Scheme for Counties [file RISA-9999-0-s001.docx]
